# Emerging concepts and challenges in the development of disease-modifying osteoarthritis drugs – a more refined perspective

**DOI:** 10.1007/s12272-025-01551-3

**Published:** 2025-06-28

**Authors:** Zsuzsa Jenei-Lanzl, Svenja Maurer, Rolf E. Brenner, Frank Zaucke, Michael Fuchs, Jana Riegger

**Affiliations:** 1 Dr Rolf M. Schwiete Research Unit for Osteoarthritis, Department of Trauma Surgery and Orthopedics, Goethe University Frankfurt, University Hospital, 60528 Frankfurt/Main, Germany; 2 Division for Biochemistry of Joint and Connective Tissue Diseases, Department of Orthopaedics, University of Ulm, 89081 Ulm, Germany; 3 Department of Orthopaedic Surgery, University of Ulm, 89081 Ulm, Germany

**Keywords:** Osteoarthritis, Disease-modifying osteoarthritis drugs, Drug development, Clinical trials, Animal models, Endotypes

## Abstract

Osteoarthritis (OA) is the most common joint disease worldwide. Despite significant efforts byresearchers, no disease-modifying osteoarthritis drugs (DMOADs) have been approved yet. This review compares preclinical and clinical studies of promising therapeutic approaches to gain insights into the potential reasons for their failure in clinical trials. For this purpose, prime examples of different therapeutic groups, including the antioxidant NAC, senotherapeutic UBX0101, anti-inflammatory drug Anakinra®, Wnt inhibitor Lorecevivint®, chondroanabolic growth factor Sprifermin™, and various protease inhibitors, are discussed in detail. The limitations of commonly used OA animal models are elaborated to understand this failure better. Moreover, this review addresses the challenges of patient stratification into different endotypes and phenotypes, the consideration of subgrouping in clinical trials, and the lack of suitable clinical outcome parameters. In summary, this review highlights potential reasons for the high failure rate of DMOADs in clinical trials and outlines key points for future improvement.

## Introduction

Osteoarthritis (OA) is a multifactorial, degenerative joint disease affecting almost 15% of the global population over 30 years of age. Strikingly, a recent study reports that about 52% of the patients suffering from OA in 2019 were below 55 years and thus of working age (Weng et al. 2024). Due to the demographic change, increase in life expectancy, and elevated obesity rates in industrial countries, the incidence of OA and thus its socioeconomic burden are expected to rise steadily (Collaborators 2023). In addition to aging, sex, lifestyle (e.g., diet, smoking), and genetic predisposition, physically demanding occupations and preceding joint injuries are considered central risk factors. Injury-related OA is often referred to as posttraumatic OA (PTOA), a special form that can even develop at a young age and accounts for about 12% of all OA cases (Riegger and Brenner 2020).

**Fig. 1:**
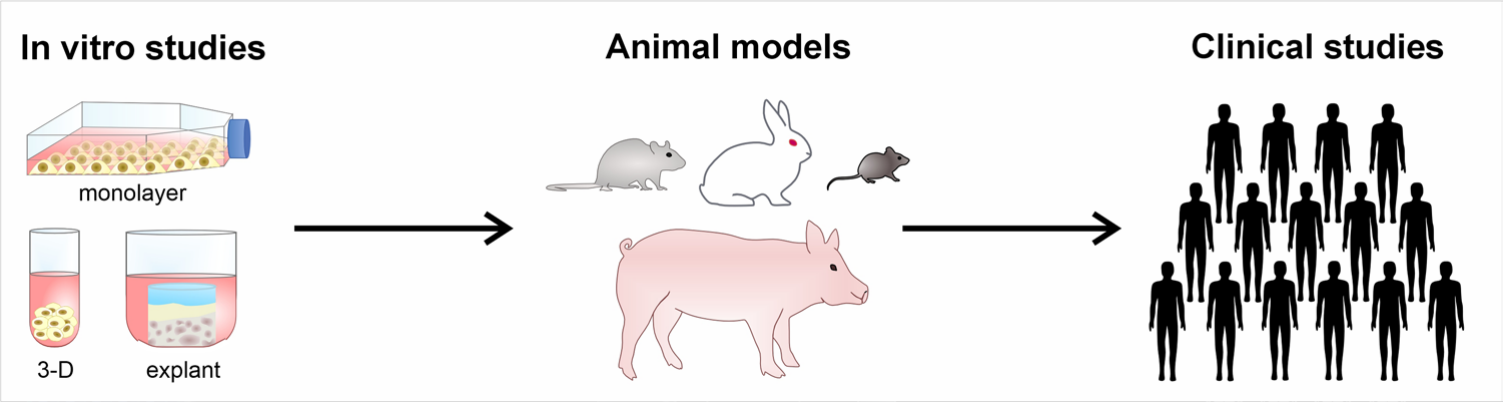
Theoretical procedure of drug development. Theoretically, a candidate potential drug is initially tested in vitro, followed by a preclinical validation in vivo. Subsequently, the compound undergoes testing in a clinical trial in patients, selected by pre-defined criteria to ensure a preferably homogenous study cohort

Non-pharmacological strategies to manage OA include weight management, physical therapy, and surgery. Pharmacological therapies are still limited to symptomatic treatment of pain and inflammation, predominantly provided by non-steroidal anti-inflammatory drugs (NSAIDs). Pain relief might help to cope with the disease for a specific time, but it neither stops OA progression nor prevents subsequent worsening of the symptoms. Besides analgesics, disease-modifying osteoarthritis drugs (DMOADs) represent a further pharmacological approach. DMOADs are developed to directly target mediators, such as cytokines, proteases, and reactive oxygen species (ROS), which promote the pathogenesis of OA, including inflammation, disruption of chondrocyte metabolism, and oxidative stress (Riegger and Brenner 2020). Although considerable progress has been made towards understanding the complex pathomechanisms of OA, no DMOADs have been approved yet by regulatory agencies, and OA is still considered an incurable disease (Rodriguez-Merchan 2023). This implies that the previous procedure of drug development (Fig. 1) might not account for all aspects of OA as a complex disease.

In this narrative review, we first provide an overview of existing DMOADs addressing different aspects of OA pathogenesis. The discussed DMOADs have been considered promising according to the outcomes of preclinical studies, but ultimately failed in clinical trials. To determine the underlying reasons for unsuccessful DMOAD development, we will (i) outline the differences between preclinical and clinical findings in more detail, (ii) discuss advantages and flaws of commonly used animal OA models for DMOAD testing, (iii) elaborate on the problematic nature of classifying OA patients into phenotypes and/or endotypes, and (iv) point out the lack of suitable and standardized clinical outcome parameters. Furthermore, we will demonstrate advances in personalized medicine in OA, which might help to improve the success of DMOADs in clinical trials.

## Inconclusive outcome of promising DMOADs in clinical trials

Regarding the enormous socioeconomic burden of OA and the suffering of patients from pain and disability, there is an urgent need for effective drug development. As mentioned above, the pathogenesis of OA is exceedingly complex and affects different joint tissues, including articular cartilage, synovium, infrapatellar fat pad, subchondral bone, as well as ligaments and tendons (Loeser et al. 2012). However, current research and drug development mainly aim at cartilage preservation or even regeneration. The disturbance of chondrometabolism is considered a consequence and a cause of cartilage degeneration. It includes multifaceted processes, such as compromised chondroanabolism, enhanced catabolic activity, and aberrant activation of the Wnt/β-catenin pathway. By addressing these pivotal pathomechanisms involved in OA development, DMOADs are expected to restore cartilage function or delay disease progression in the affected joint (Oo et al. 2021; Kim et al. 2022). Despite an auspicious outcome from preclinical studies with various DMOADs addressing different key mediators in OA, respective clinical studies have mainly led to disappointing results.

In the following paragraphs, we provide an overview of the clinical performance of DMOADs, focusing on the currently most discussed candidates, which are additionally summarized in Table 1. The preclinical outcome of these DMOADs is compiled in Table 2.

**Table 1:** Summary of current clinical trials of DMOADs, their outcome, and potential reasons for failure

Drug Class	Compound	Phase	Outcome/potential reason for failure	Trial number/references
Antioxidant	NAC	Pilot study	No placebo group Retrospective cohort study—oral intake of NAC (i.a. concentration unclear)	(Ozcamdalli et al. 2017) (Yeh et al. 2020)
Senolytic	UBX0101	Phase 2	Strong response in placebo group; only single i.a. injection; no hit-and-run approach	NCT04129944 NCT04349956 (Lane et al. 2021)
Anti-inflammatory drug	IL-1RA Anakinra®	Phase 2	Strong response in placebo group; only single i.a. injection; short duration of effect	NCT00110916(Chevalier et al. 2009)
Wnt inhibitor	Lorecivivint® SM04690	Phase 2a Phase 3	Primary endpoint not achieved, but identification of a subpopulation with stronger response First results: Primary endpoint not achieved; only single i.a. injection; late intervention (advanced OA patients) better results in patients with less severe structural disease; during COVID pandemic (impact unknown)	NCT02536833 (Yazici et al. 2020) NCT04385303 NCT03928184 NCT04520607 NCT05603754 (Tambiah et al. 2024) (Swearingen et al. 2025) (Swearingen et al. 2024)
Protease inhibitors	ADAMTS-5 inhibitor/ S201086 GLPG1972 MMP inhibitor PG116800 PG530742	Phase 2	Primary endpoints not achieved; no effect vs. placebo; no significant reduction in cartilage; loss and no modification of symptoms musculoskeletal side effects compromise the safety of long-term systemic administration	NCT03595618 (Schnitzer et al. 2023) NCT00042156 (Krzeski et al. 2007)
Growth factor	rhFGF18 Sprifermin™	Phase 2 Phase 3	Post-hoc analysis suggests that Sprifermin™ should be evaluated further in clinical trials; long-term structural modification of articular cartilage was maintained with Sprifermin™ versus placebo Ongoing	NCT01919164 (Hochberg et al. 2019) (Eckstein et al. 2021)

*ADAMTS* a disintegrin and metalloproteinase with thrombospondin motifs, *i.a* intra articular, *IL-1RA* interleukin 1 receptor antagonist, *MMP* matrix metalloproteinase, *NAC* N-acetyl cysteine, *rhFGF18* recombinant human fibroblast growth factor 18

**Table 2:** Overview of the respective preclinical studies

Therapy	Type of OA	Animal/Joint/Model	Outcome	Reference
**Antioxidants** NAC	Age-related/natural OA natural ageing (different strains)	Rat Femoral head wildtype	Reduced ROS levels in old chondrocytes only (ROS increased with age)	(Jallali et al. 2005)
	PTOA	Rat Knee ACLT	Inhibited chondrocyte apoptosis and articular cartilage degeneration	(Nakagawa et al. 2010)
		Rat Knee MML excision + bilateral meniscectomy + MCL, ACL, and lateral extensor tendon dissection	Reduced MMP-13 expression and down-regulation of collagen type II in chondrocytes as well as chondrocyte apoptosis were inhibited	(Kaneko et al. 2019)
		Lapine Knee blunt single-impact cartilage trauma	Reduced trauma-induced hypocellularity, MMP-13 expression, and cell cluster formation; enhanced synovial concentrations of CPII and cartilage proteoglycan staining intensity	(Riegger et al. 2019)
		Porcine Knee, intra-articular fracture	Protected against PTOA at 6 months, including maintenance of proteoglycan content; decreased histological disease scores, and normalized chondrocyte metabolic function	(Coleman et al. 2018)
	Chemically induced	Rat TMJ H_2_O_2_ and ferrous iron (components of the Fenton reaction which generate free radicals)	Prevented carbonyl group formation—an indication of oxidative stress causing synovial lining hyperplasia, fibrous intima matrices, fibrous subintimal tissues, hypervascularity, inflammatory infiltration, perivascular fibrosis, myxoid degeneration, and erythrocyte extravasation	(Sheets et al. 2006)
	Genetically modified	Murine Knee cartilage-specific SEPHS1-KO	Aging-associated and post-traumatic OA rescued—reduced OARSI, synovitis, osteophyte formation and pain (weight bearing analysis)	(Kang et al. 2022)
**Senotherapeutics** UBX0101	PTOA	Murine Knee ACLT	Attenuated OA development (cartilage), reduced pain	(Jeon et al. 2017)
			Diminished response to UBX0101 treatment (lower OARSI score) in aged mice compared to their younger counterparts Removal of senescent cells and the correlative reduction of oxidative stress are insufficient to fully drive reversal of OA pathology in advanced age	(Faust et al. 2020) (Chin et al. 2023)
fisetin (flavonoid, senolytic)		Murine Knee DMM	Less cartilage destruction and lower OARSI scores; Reduced subchondral bone plate thickness and alleviated synovitis	(Zheng et al. 2017)
**Anti-inflammatory drugs** IL-1RA (Anakinra®) IL-1RA (Anakinra®)	PTOA	Rat Knee intra-articular fracture	Reduced CTX-II in cartilage but also GAG, TRAP in bone, serum pro-inflammatory cytokines; increased synovial fluid MCP-1, MCP-3, MIP2) with concomitant decrease in IL-17A	(Valerio et al. 2023)
		Rat Knee ACLT	Improved lubricin expression, reduced cartilage and synovial scores	(Elsaid et al. 2015)
anti-TNF antibody adalimumab infliximab	PTOA	Rat Knee ACLT	reduced severity of cartilage lesions (lower Mankin score), reduced destruction of subchondral trabecular microstructure & decreased MMP-13 expression	(Ma et al. 2015)
		Lapine knee Hulth technique (cruciate ligaments & medial menisci resection	Significantly lower Mankin scores, lower synovial fluid TNF and NO concentrations (IL-1β unchanged)	(Zhang et al. 2012)
infliximab		Rat Knee intra-articular fracture	Only minimal positive effect of healing (but strong side effects: Liver and metabolic dysfunction)	(Valerio et al. 2023)
**Wnt-Inhibitors** Lorecivivint® (SM04690)	PTOA	Lapine and rat TMJ partial TMJ discectomy	Protected condylar cartilage from degeneration and attenuated abnormal subchondral bone remodeling	(Hua et al. 2022)
		Rat Knee ACLT + pMMx	Increased cartilage thickness & protection from cartilage catabolism resulting in significantly improved OARSI scores	(Deshmukh et al. 2018)
	Chemically induced	Rat Knee MIA-iNduced	Inhibited production of inflammatory cytokines and cartilage degradative enzymes, resulting in increased joint cartilage; decreased pain, and improved weight-bearing function	(Deshmukh et al. 2019)
**Protease inhibitors** MMP-13 inhibitor **PF152**	PTOA	Canine Knee mensiectomy	Reduced the severity of cartilage lesions; decreased levels of collagen II and aggrecan degradation products	(Settle et al. 2010)
ADAMTS-5 inhibitor GLPG1972/S201086	PTOA	Rat Knee meniscectomy	Significantly reduced in OARSI score and cartilage fibrillation; Prevented proteoglycan loss and subchondral bone plate thickening	(Lepescheux et al. 2018)
ADAMTS-4/−5 inhibitor AGG-523	PTOA	Rat Knee meniscal tear procedure	Reduced the release of aggrecanase-generated aggrecan fragments into rat joints	(Chockalingam et al. 2011)
**Chondroanabolic** FGF-18/Sprifermin™	PTOA	Murine Knee DMM	Inhibited proteoglycan loss, lower OARSI scores	(Briat et al. 2022)
		Rat Knee meniscal tear procedure	Dose-dependent increases in neocartilage formation, resulting in significant reductions in cartilage degeneration scores; increased remodelling of subchondral bone	(Moore et al. 2005)
		Rat Knee meniscal tear procedure	Prevented cartilage degeneration	(Mori et al. 2014)

*ACLT* anterior cruciate ligament transection, *CPII* type II procollagen carboxy-propeptide; *CTX-II* cross-linked C-telopeptide of type II collagen, *DMM* destabilization of the medial meniscus, *MCP-1/−3* Monocyte chemoattractant protein-1/−3, *MIA* monosodium iodoacetate, *MIP2* macrophage inhibitory protein 2, *MML* medial meniscotibial ligament, *pMMx* partial medial meniscectomy*, SEPHS1* selenophosphate synthetase 1, *TMJ* temporomandibular joint

### Oxidative stress – antioxidants

Oxidative stress can result from the imbalance between ROS production and its elimination through the cellular antioxidant defense system. Increasing oxidative stress is a major driver of OA progression by activating several redox-sensitive pro-inflammatory signaling pathways like NF-κB and induction of mitochondrial dysfunction, apoptosis, and cellular senescence in chondrocytes (Riegger et al. 2023b). ROS promote cartilage degradation, impair matrix synthesis, and enhance subchondral bone sclerosis (Zahan et al. 2020; Tudorachi et al. 2021). A large amount of research has been carried out on antioxidants of natural origin as potential treatments for OA, such as resveratrol, curcumin, oleuropein, quercetin, pomegranate extract, rosmarinic acid, and epigallocatechin-3-gallate – to name but a few. Additionally, most polyphenols and flavonoids are known for their anti-inflammatory effects in OA, as previously reviewed elsewhere (Deligiannidou et al. 2020; Tudorachi et al. 2021).

**Fig. 2:**
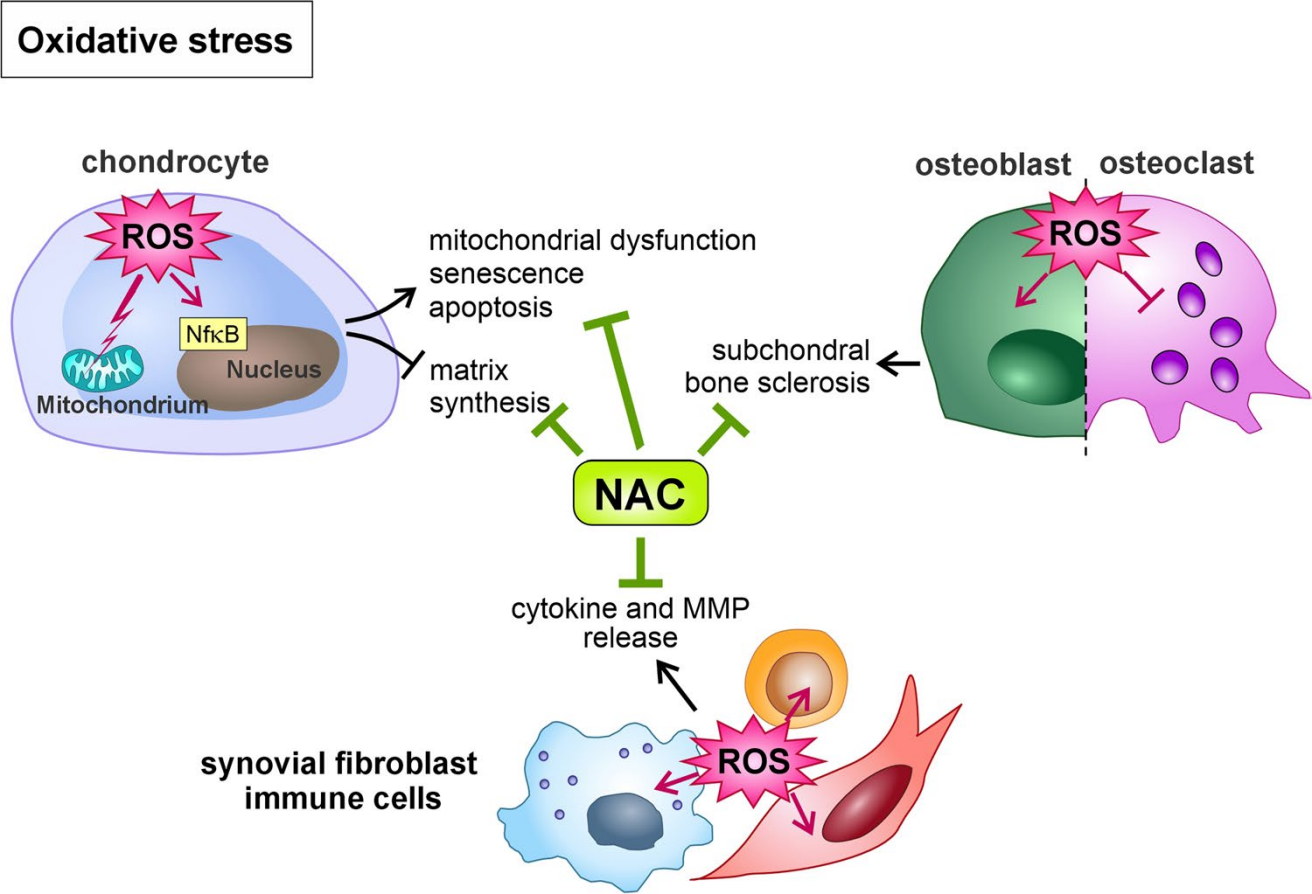
Tackling oxidative stress by the antioxidant N-acetyl cysteine (NAC). As a crucial driver of OA, oxidative stress has detrimental effects on tissue homeostasis. The antioxidant NAC efficiently eliminates ROS in various joint cell types, thus attenuating pathophysiologic processes, including cell death, subchondral sclerosis, inflammation, and catabolic enzyme expression. Overall, reducing oxidative stress facilitates normal cell function and therefore pro-regenerative processes. Please see the main text for further details

A key component of the cellular antioxidant defense system is glutathione (GSH), a tripeptide consisting of glutamate, glycine, and cysteine (Forman et al. 2009). As a synthetic derivative of cysteine, N-acetyl cysteine (NAC) represents a precursor for GSH de novo synthesis, but can also directly scavenge ROS through its thiol group (Samuni and Goldstein 1830). Hence, NAC targets various ROS-mediated pathomechanisms in OA (Fig. 2) and has been tested in multiple animal models of PTOA as outlined in Table 2.

In a pilot study, the effect of a single intra-articular (i.a.) NAC or hyaluronic acid (HA) injection was compared in 20 participants with primary knee OA (≥ 40 years, Kellgren-Lawrence (KL) grade 2–3). A significant reduction of the total Western Ontario and McMaster Universities Arthritis Index (WOMAC) score, total oxidant status, and synovial matrix metalloproteinase (MMP)−3 concentration was demonstrated in both treatment groups. Moreover, the NAC group displayed a significant decrease in serum chondroitin 6-sulfate and cross-linked carboxyl terminal telopeptide of collagen type II (CTX-II) levels in the synovial fluid. Altogether, NAC tended to achieve better results than HA in this study. However, it must be considered that only NAC and HA were compared, and no placebo group was included (Ozcamdalli et al. 2017). In contrast, another clinical study demonstrated a connection between oral intake of NAC and an increased risk of developing knee OA. This retrospective study was performed in a Taiwanese cohort (12,928 NAC users; 51,715 nonusers, > 25 years) with patients who received NAC orally for over 28 days within one year (Yeh et al. 2020).

Despite the positive reports on the therapeutic efficacy of NAC in the context of OA, the antioxidant has not yet been accepted as DMOAD.

### Cellular senescence – senotherapeutics

Cellular senescence is essential in developing many age-related diseases, including OA. Senescent cells (SnCs) are characterized by cell cycle arrest, apoptosis resistance, and the so-called senescence-associated secretory phenotype (SASP) (Childs et al. 2015; Jeon et al. 2018). The latter refers to the elevated secretion of pro-inflammatory cytokines and chemokines as well as catabolic enzymes, which drive the progression of OA and may also negatively affect neighboring cells in a paracrine manner (Jeon et al. 2018). Targeting cellular senescence can be achieved by two different groups of senotherapeutics: senomorphics that mainly act via inhibition or neutralization of SASP factors and senolytics, which selectively eliminate SnCs via apoptosis (Coryell et al. 2021; Mylonas and O’Loghlen 2022). Senomorphics include direct inhibitors of specific SASP factors (e.g., tocilizumab: interleukin-6 (IL-6) neutralizing antibody (AB); etanercept: TNF neutralizing AB) as well as inhibitors of intracellular pathways (e.g., vitexin: NF-κB inhibitor, ruxolitinib: JAK1/2 inhibitor) (Zhang et al. 2021; Chaib et al. 2022). Therefore, senomorphics alleviate the harmful effects of SnCs only as long as the drug is applied, while senolytic clearance of SnCscontinuously reduces SASP factor secretion.

**Fig. 3:**
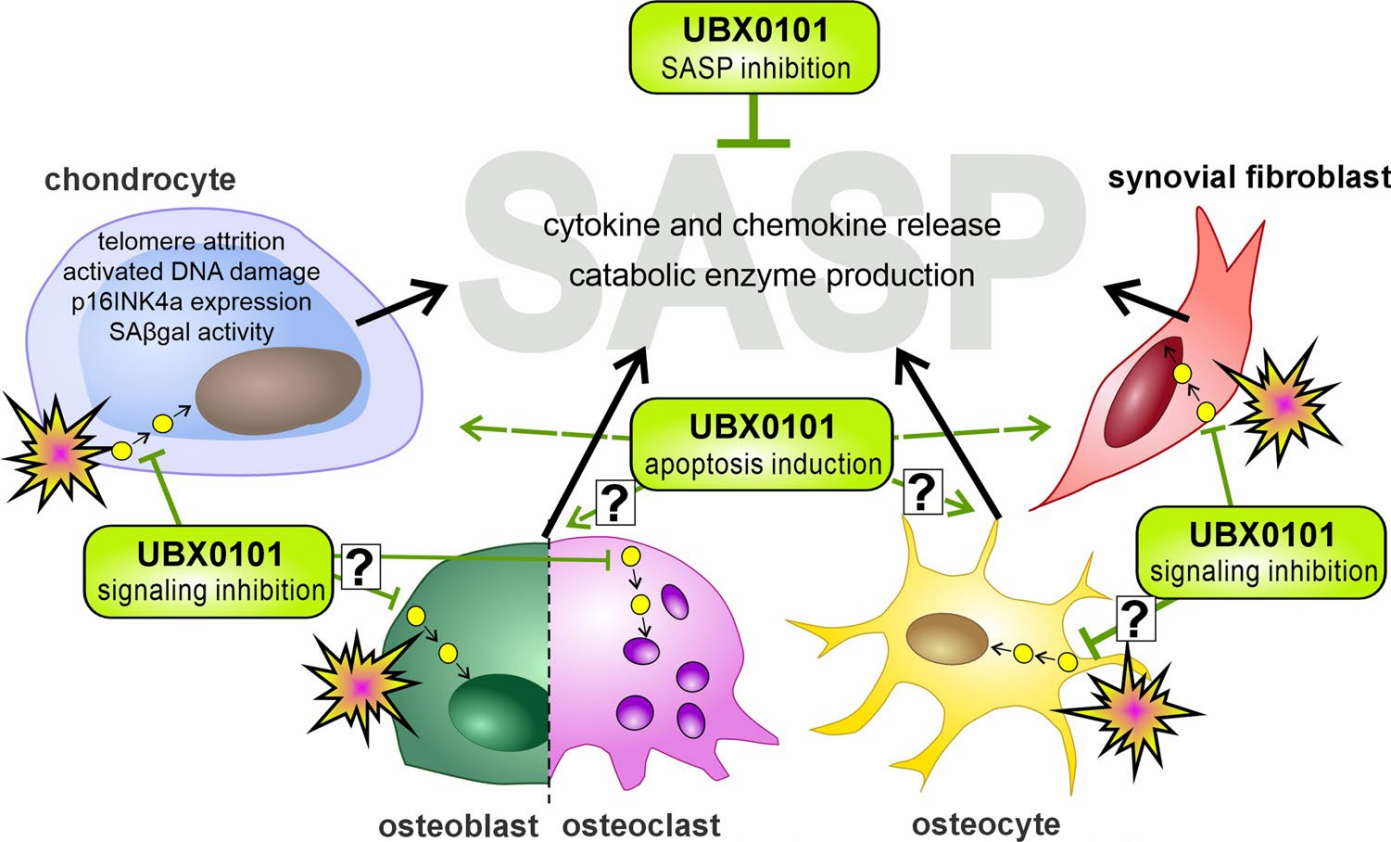
Tackling cellular senescence by the senolytic UBX0101. Oxidative stress and chronic low-grade inflammation result in the accumulation of senescent cells in joint-related tissues. Due to the excessive release of catabolic and pro-inflammatory mediators, senescence substantially contributes to OA progression. Senescent cells can be selectively eliminated using senolytics, such as UBX0101, thus attenuating the secretion of SASP factors and consequent tissue degeneration and inflammation. While the efficient clearance of dysfunctional chondrocytes and synovial cells has been intensively studied, the therapeutic effects of senolytics on subchondral bone remodeling during OA development must be determined (McCulloch et al. 2017)

Various senolytics, including inhibitors targeting BCL-2 family proteins, tyrosine kinases, HSP90, and p53 inhibitors, have been tested in different in vitro and in vivo models. The senolytic UBX0101 (outlined in Fig. 3) is a p53/MDM2 interaction inhibitor and advanced to clinical trials but failed in phase 2 (Zhang et al. 2021). Before the clinical trials, Jeon et al. tested UBX0101 in vitro and multiple OA mouse models for its effectiveness, as described in Table 2. The in vivo experiments made clear that at least five to six injections were necessary and that no long-lasting effects of the therapy could be achieved (Jeon et al. 2017). The phase 1 clinical trial with UBX0101 in 48 participants (40–85 years, KL grade 1–4, numeric rating scale (NRS) daily pain 4–9), with single i.a. doses of 0.1–4.0 mg displayed meaningful results, especially for the highest concentrations demonstrating beneficial effects regarding the WOMAC subscores pain and function. In addition, a dosage of up to 4.0 mg was well tolerated, and no serious adverse events were observed (NCT03513016) (Hsu et al. 2020). However, the results from phase 1 could not be confirmed in the phase 2 study (183 participants, KL grade 1–4, NRS daily pain 4–9). The reason for unsatisfactory results was the pronounced response in the placebo group, which made it difficult to attribute the observed effects to the UBX0101 treatment (NCT04129944) (Lane et al. 2021). Most likely, multiple injections of UBX0101 might have been required, as previously applied in preclinical studies. Moreover, the so-called hit-and-run treatment – an intermittent administration regimen – has been described as more effective than a continuous application or single doses of senolytics (Kirkland and Tchkonia 2020). Accordingly, the hit-and-run approach was chosen in a current clinical study testing the senolytic drug fisetin that was orally administered to OA patients (40–80 years, KL grade 2–4, pain NRS 4–10) (NCT04210986). The therapeutic effects of fisetin in OA were previously demonstrated in a mouse model with surgical destabilization of the medial meniscus (DMM), in which daily administration of the drug resulted in a lower levels of cartilage degeneration and Osteoarthritis Research Society International (OARSI) scores as well as in reduced synovitis and subchondral bone plate thickening (Zheng et al. 2017).

### Inflammation – anti-inflammatory drugs

OA is characterized by chronic low-grade inflammation, with the interaction between synovium and cartilage tissue playing a significant role. Infiltration of macrophages into the synovial membrane and their activation by the pathological microenvironment induce the secretion of pro-inflammatory cytokines and MMPs (Terkawi et al. 2022). IL-1β, IL-6, and TNF are the most abundant cytokines, but IL-2, −8, −15, −17, −18, and −21 contribute to pro-inflammatory processes (Kapoor et al. 2011; Miller et al. 2014). IL-1β, IL-6, and TNF are known for their catabolic effects mediated by the elevated production of MMPs in chondrocytes (Molnar et al. 2021). Above all, TNF and IL-1β induce the release of further pro-inflammatory cytokines. Due to their pro-analgesic effects, these cytokines directly promote chronic OA pain by lowering the sensory neuron threshold in the joint and the dorsal root ganglia (Miller et al. 2014).

**Fig. 4:**
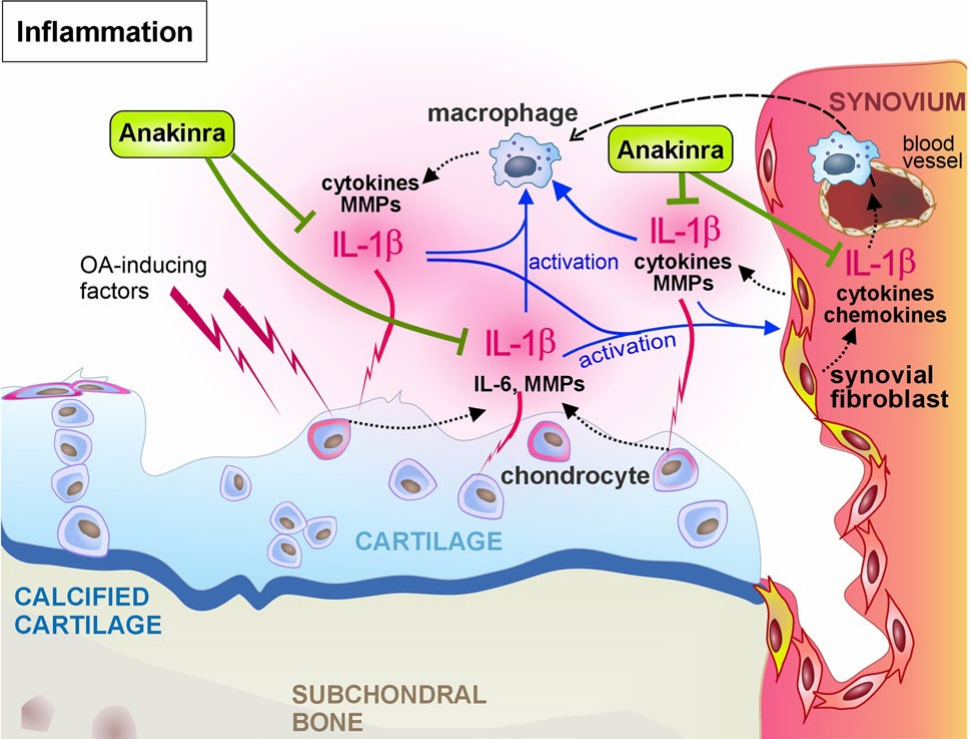
**Tackling inflammation by the anti-inflammatory drug Anakinra**^.^ Pro-inflammatory cytokines, including IL-1β, mainly derive from the synovium in the synovial joint. Besides cytokine-directed antibodies, inhibition of cytokine receptors represents a promising therapeutic option to reduce the detrimental effects on cartilage integrity. One well-studied example of this approach is the treatment with recombinant IL-1RA, Anakinra®. Please see the main text for further details

In recent years, various cytokine-targeting therapies have been investigated, such as the anti-TNF antibodies infliximab and adalimumab or the recombinant IL-1R antagonist (IL-1RA) Anakinra® (Kapoor et al. 2011). The therapeutic effects of Anakinra® are illustrated in Fig. 4. Over the years, extensive research has been conducted on IL-1RA as a therapeutic agent for rheumatoid arthritis (RA), with clinical trials demonstrating positive results (Furst 2004). Kineret (Anakinra®) is FDA-approved for treating RA but not OA. Although IL-1RA exhibited therapeutic effects in OA animal models, as described in Table 2 in detail, Anakinra® did not pass phase 2 in clinical trials (Chevalier et al. 2009). In a phase 1 safety study, Anakinra® (150 mg) improved pain and the WOMAC global score in 13 patients. The treatment was well tolerated, and no acute inflammatory response was observed (Chevalier et al. 2005). However, in the phase 2 clinical study, which included 170 subjects (160 completed the study, ≥ 18 years, exclusion criteria: secondary OA and KL grade 4) no significant differences were identified for 50 mg or 150 mg Anakinra® in the WOMAC pain, function and stiffness score (week 4) compared to the placebo group. This was partly due to the strong response in the placebo group. However, on day 4 after injection, significant pain reduction was observed in the 150 mg group, suggesting that the drug exerts at least short-term effects. This is in line with the short plasma half-life of the drug (~ 4 h). Higher drug concentrations may be needed, but 150 mg was already set as the maximum tolerable dosage. Alternatives are repetitive injections, a more potent IL-1RA, or administration of a gene therapeutic approach (Chevalier et al. 2009) (NCT00110916).

A possible reason why Anakinra® is effective in RA but not in OA is that the IL-1 pathway has not been confirmed as a key mediator in the pathogenesis of OA. However, some patients exhibit elevated synovial IL-1β levels. Thus, the cytokine has overall been controversially discussed as a potential therapeutic target in OA (Vincent 2019). Nevertheless, clinical application of the IL-1β antibody canakinumab reduced pain in knee OA patients with increased baseline hsCRP levels. Therefore, considering endotypes is necessary, and anti-inflammatory therapy could benefit specific patient subgroups (Conaghan et al. 2021).After promising preclinical studies (Table 2), the first clinical trials on IL-1RA gene therapy have been initiated using Sc-rAAV2.5IL-1Ra (NCT02790723) or FX201 (NCT04119687).

### Wnt signaling – Wnt inhibitors

**Fig. 5:**
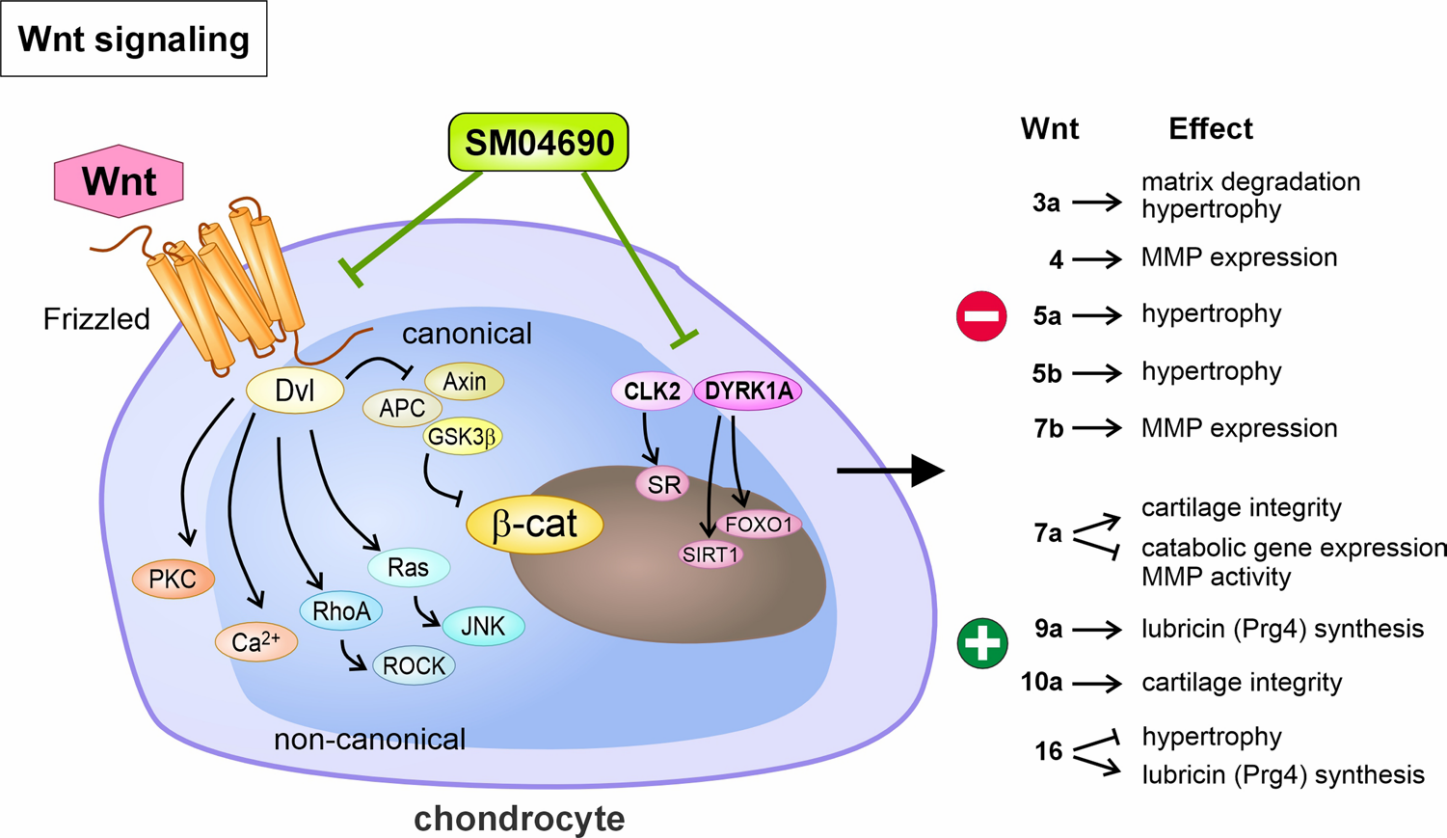
Tackling Wnt signaling by the Wnt inhibitor SM04690. The Wnt signaling pathway plays an essential role in cartilage homeostasis by modulating the chondrocyte phenotype in various ways, depending on the Wnt protein and accordingly on the subsequent canonical or non-canonical signaling pathways. Inhibition of the Wnt signaling, e.g., by SM04690, represents a potential therapeutic option in OA. Please see the main text for further details

The Wnt signaling pathway plays an essential role in OA’s pathogenesis by inducing unfavorable and protective processes. The canonical β-catenin-dependent pathway of gene expression regulation is based on the inhibition of the β-catenin-degrading enzyme glycogen synthase kinase 3β (GSK3β) upon ligand binding. In contrast, the non-canonical pathway is linked to increased intracellular calcium and activation of other kinases. The balance between both pathways is of substantial importance for tissue homeostasis (Stampella et al. 2017; Lories and Monteagudo 2020). Which pathway becomes predominantly activated – and thus the nature of mediated effects – depends on the targeted receptor subtype (Cheng et al. 2022; Li et al. 2023a). Several modulators strongly regulate Wnt signaling, which is essential, considering both inactivation and overexpression of its effector protein β-catenin promote OA. Wnt16, for example, is crucial to lubricin (proteoglycan 4) synthesis in the superficial cartilage layer (Nalesso et al. 2017). In contrast, overactivation of Wnt signaling is associated with hypertrophic differentiation of chondrocytes, calcification, subchondral bone remodeling, osteophyte formation, synovial inflammation, and thus, OA progression (Stampella et al. 2017; Lories and Monteagudo 2020). Figure 5 provides a brief overview of the effects of different Wnt proteins, as also reviewed elsewhere (Li et al. 2023a).

Deshmukh et al. identified a small molecule Wnt inhibitor (SM04690, Lorecivivint®, adavivint) via high-throughput screening and demonstrated its therapeutic efficacy in OA animal models (Table 2**, **Fig. 5) (Deshmukh et al. 2018). Moreover, a new mechanism of Lorecivivint®-mediated modulation of the Wnt signaling pathway was reported through the inhibition of CDC-like kinase two and dual-specificity tyrosine phosphorylation-regulated kinase 1 A, which are associated with inflammation and impaired chondrogenesis (Deshmukh et al. 2019). In a phase 1 clinical trial study (61 participants, primary knee OA, 50–75 years, KL grade 2–3), Lorecivivint® (single i.a. of 0.03 mg, 0.07 mg, 0.23 mg, or placebo) was safe, well-tolerated, and improved the total WOMAC score as well as the WOMAC function and pain sub-scores, physician global assessment (MDGA), Pain Visual Analogue Scale score, and Outcome Measures in Rheumatology-Osteoarthritis Research Society International (OMERACT-OARSI) (Yazici et al. 2017) (NCT02095548). Nevertheless, the primary endpoint was not achieved in a phase 2a clinical trial (455 participants, primary knee OA, 40–80 years, KL grade 2–3). Still, a subpopulation with a stronger response to the therapy was identified. The better outcome of the unilateral symptomatic knee OA group regarding pain was explained by the possibly easier discrimination of the pain in the affected knee from other pain sources. In addition, the authors assumed that the significant increase in radiographic medial joint space width in this subgroup results from biomechanical asymmetries, as previously described in unilateral knee OA (Yazici et al. 2020) (NCT02536833). After completion of the phase 2b clinical trial (Yazici et al. 2021) (NCT03122860) and its post hoc analysis (Tambiah et al. 2021), several phase 3 clinical trials with the optimally determined concentration of 0.07 mg Lorecivivint® are ongoing (40–80 years, KL grade 2–3) (NCT04520607; NCT05603754; NCT04385303; NCT03928184).

### ECM degeneration – protease inhibitors

**Fig. 6:**
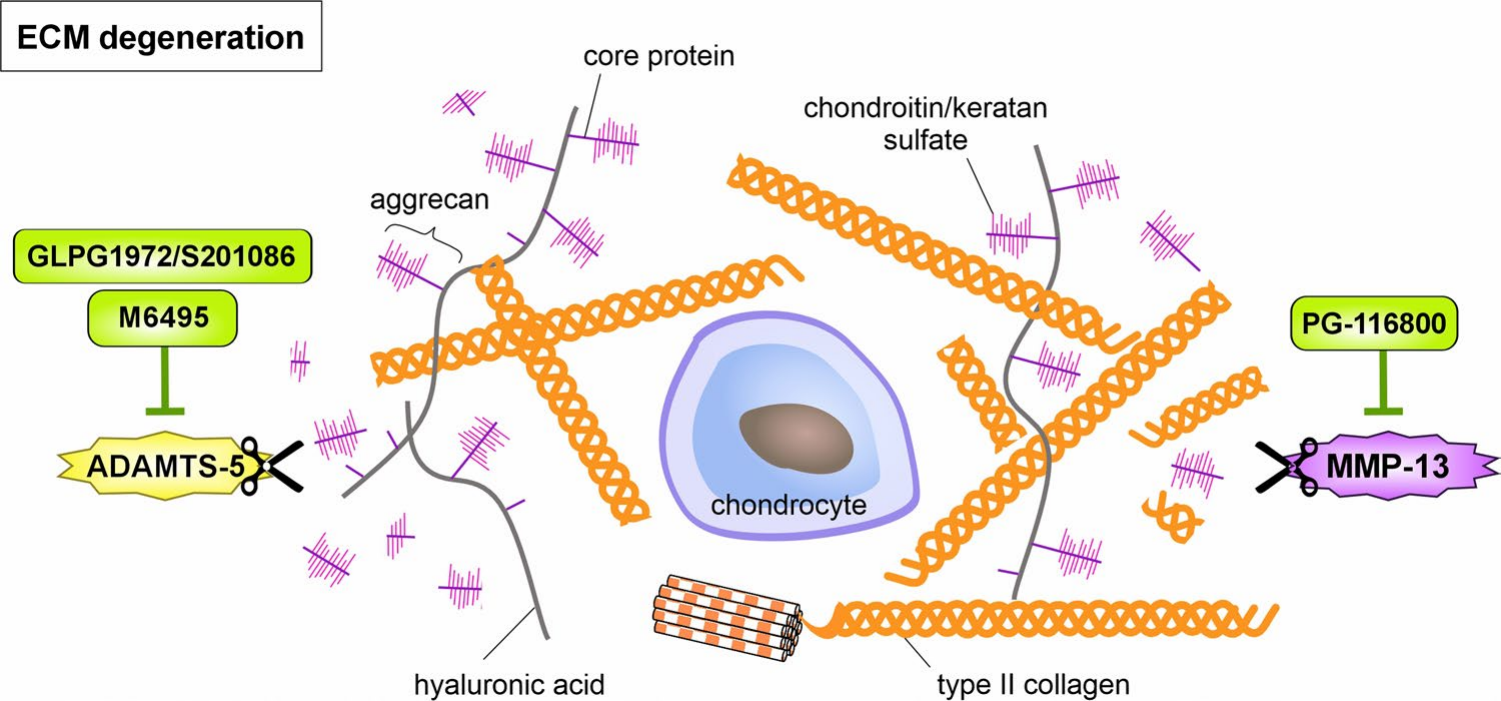
Tackling catabolism by protease inhibitors. Different MMPs and ADAMTS mainly drive matrix degradation. In the context of OA, MMP-13 and ADAMTS5 represent the predominant proteases causing proteolytic cleavage of type II collagen and aggrecan, the main components of hyaline cartilage. Accordingly, application of protease inhibitors, such as GLPG1972/S201086 and M6495 (ADAMTS-5 specific) as well as PG-116800 (broader MMP inhibitor also inhibiting MMP-13), exerts chondroprotective effects and is thus considered a potentially suitable treatment in OA. Please see the main text for further details

In degenerative disorders, including OA, the degradation of the extracellular matrix (ECM) by proteases is a common feature (Grillet et al. 2023). Collagen type II and aggrecan are the most abundant components of the cartilage ECM (Grossmann and Grossmann 1968), and, therefore, it is pretty apparent to consider inhibitors of the major degrading proteases as potential DMOADs. Collagens are mainly cleaved by members of the MMP family, while aggrecan is predominantly degraded by aggrecanases of the a disintegrin and metalloproteinase with thrombospondin motifs (ADAMTS) family. In cartilage, and in particular during the progression of OA, MMP-13 (also called collagenase-3) and ADAMTS-5 are generally accepted as the most relevant matrix-degrading enzymes (Ashruf and Ansari 2022). The vital contribution of these two proteases to OA development was confirmed in several genetically modified mouse strains. Mice lacking MMP-13 showed less cartilage erosion after DMM than controls (Little et al. 2009). Cartilage-specific expression of a constitutively active MMP-13 resulted in a joint pathology similar to OA (Neuhold et al. 2001). Also, in mice deficient for ADAMTS-5, DMM surgery led tosignificantly reduced cartilage degradation compared to control animals (Hudson et al. 2006). Overall, the proteases MMP-13 and ADAMTS-5 are attractive targets for OA treatment (Fig. 6).

A large body of literature is already analyzing the effects of specific protease inhibitors in vitro and animal models. However, concerns have been raised regarding the use of MMP inhibitors in humans. Musculoskeletal adverse events such as arthralgia, palmar fibrosis, persistent tendon thickness or nodules have been reported for the MMP inhibitor PG-116800 in patients with knee OA (401 patients, 40 to 80 years of age with primary knee OA according to the ACR criteria, five groups: placebo, 25, 50, 100, or 200 mg, oral intake of PG-530742, the dehydrated salt form of PG-116800, twice daily for 1 year, NCT00042156) in a dose-dependent manner (Krzeski et al. 2007). Most likely, MMPs also have essential functions in non-cartilaginous tissues, and the interference with the homeostasis of other tissues causes negative effects, as shown for PF152. This MMP-13 inhibitor reduced human cartilage degradation ex vivo and the severity of cartilage lesions caused by partial medial meniscectomy in a canine PTOA model (Settle et al. 2010) but caused nephrotoxicity through off-target effects. Nevertheless, optimizing specific small-molecule MMP-13 inhibitors (Li et al. 2011) and their delivery methods to damaged cartilage are still under investigation (Bedingfield et al. 2021).

### Aggrecanase inhibitors

As ADAMTS-5 is a key protease in cartilage degradation, the discovery of the small molecule ADAMTS-5 inhibitor GLPG1972/S201086 was an essential first step in the generation of a potential DMOAD (Brebion et al. 2021). The pharmacological characterization of this compound demonstrated its potency and selectivity in vitro. GLPG1972/S201086 inhibited human ADAMTS-5 with eightfold selectivity over ADAMTS-4 and could reduce IL-1β-induced aggrecan degradation in murine and human cartilage explants. In addition, its protective effect on cartilage and bone was demonstrated in the DMM mouse model and meniscectomized rats (Clement-Lacroix et al. 2022) (Table 2). The safety and tolerability of GLPG1972/S201086 were then tested in a first-in-human study (NCT02612246) in healthy individuals (n = 41) and later in patients (n = 30) with knee or hip OA (NCT03311009) who received single and multiple ascending oral doses (≤ 300 mg once daily for four weeks). It was shown that GLPG1972/S201086 was well tolerated at all doses and that multiple doses significantly reduced the levels of aggrecan fragments in serum samples (van der Aar et al. 2022). Based on these findings, the randomized, placebo-controlled dose-ranging trial ROCCELLA (NCT03595618) was designed to analyze the efficacy and safety of treating knee OA (PMID 36474770). However, the evaluation of this phase 2 trial with 932 participants aged 40–75 years with moderate-to-severe pain and a KL grade of 2–3 revealed no significant reduction in cartilage loss and no modification of symptoms (Schnitzer et al. 2023). Therefore, the clinical use of this inhibitor is currently not considered further. Another selective oral ADAMTS-4 and −5 inhibitor, AGG-523, reduced the levels of a specific aggrecan fragment in synovial fluid released after meniscal tear in a rat OA model (Chockalingam et al. 2011). This inhibitor then entered a phase I clinical study with 64 subjects (men and women, at least 55 years old with knee OA requiring total knee replacement surgery, 16 controls with placebo) in two randomly assigned groups, either receiving 900 mg twice or 1800 mg once a day for 4 weeks. However, the trial was suspended for unknown reasons (NCT00454298).

The anti-ADAMTS-5 inhibiting nanobody M6495 was generated parallel to the inhibitor mentioned above. M6495 dose-dependently inhibited aggrecan degradation in human ex vivo cartilage (Siebuhr et al. 2020). The safety, tolerability, and pharmacodynamics of M6495 were analyzed in two phase 1 trials in healthy subjects (a single subcutaneous injection of 1–300 mg, n = 54) and OA patients (n = 32, KL grade 2 to 4, pain > 40 on the WOMAC pain subscale, three doses every two weeks with 75–300 mg per dose) (Bihlet et al. 2024). These trials demonstrated that the nanobody was well tolerated and adequately safe. However, the clinical evaluation of its disease-modifying effects remains to be determined in future studies.

### Impaired anabolism – chondroanabolic treatment

**Fig. 7:**
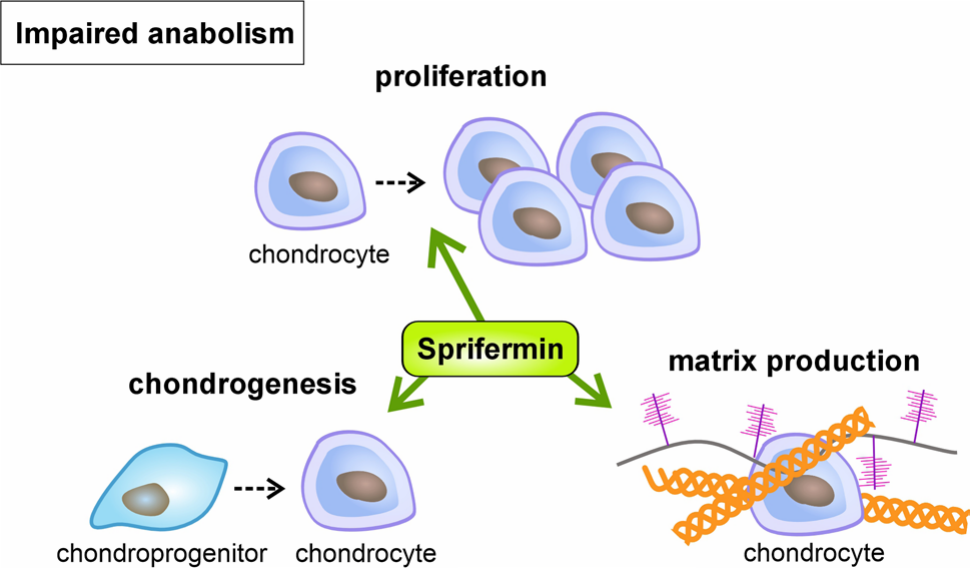
Tackling impaired anabolism by chondroanabolic growth factor 18. Recombinant human FGF18 (Sprifermin™) is OA’s first drug promoting chondroanabolic effects. The growth factor not only induces matrix production but also chondrogenesis and proliferation, which are crucial processes in terms of cartilage repair. Please see the main text for further details

Cartilage has a low intrinsic regenerative capacity. Nevertheless, promoting repair instead of preventing degradation could be an alternative approach to treat cartilage loss in OA. The most promising results regarding anabolic treatment were achieved with recombinant human FGF18 (Sprifermin™). Convincing in vitro studies have demonstrated that Sprifermin™ promotes chondrogenesis, stimulates chondrocyte proliferation, and the production of a hyaline cartilage matrix, including collagen type II (Fig. 7) (Ellsworth et al. 2002; Gigout et al. 2017). Promising results were also obtained in vivo after i.a. injection in an ovine model with chondral defects (Power et al. 2014) and a rat meniscal tear model of OA (Moore et al. 2005) (for further details, see Table 2).

These observations paved the way for clinical trials and a first randomized, double-blind, placebo-controlled trial with 192 randomized patients (age ≥ 40 years) with established knee OA according to ACR clinical and radiological criteria. Of these, 168 patients were evaluated for the primary efficacy endpoint: the change in central medial femorotibial compartment cartilage thickness at 6 and 12 months after a single i.a. Sprifermin™ injection. The results showed that Sprifermin™ injection (10, 30, and 100 µg) was not associated with local or systemic safety concerns (Lohmander et al. 2014). In addition, a dose-dependent effect of i.a. Sprifermin™ injections on cartilage thickness across the femorotibial joint and on joint space narrowing was observed in knees treated with the highest dose of 100 µg. Further post hoc analyses suggested that this concentration benefits bone marrow lesions but did not reduce synovitis (Eckstein et al. 2015; Roemer et al. 2016). This was followed by the multicenter, randomized, double-blind, placebo-controlled, parallel-group phase 2 trial FORWARD (NCT01919164). Patients were aged between 40 and 85 years with symptomatic, radiographic knee OA and a KL grade of 2 or 3. 549 participants in 5 randomized groups received placebo, 30 or 100 µg Sprifermin™ every 6 or every 12 months and the primary endpoint “change in cartilage thickness” was measured by magnetic resonance imaging (MRI) at 2 years. After two years, the i.a. injection of 100 µg SpriferminTM significantly improved cartilage thickness (Hochberg et al. 2019). According to 5-year results from the FORWARD trial with 378 remaining patients, Sprifermin™ is the first DMOAD that promotes regeneration of damaged articular cartilage (Eckstein et al. 2021). However, a recent study reported that different subgroups in the investigated cohort respond differently depending on the serum marker levels, indicating collagen type II formation, as further described below (Bay-Jensen et al. 2022).

In summary, the long-term benefit of Sprifermin™ is uncertain, and more studies are needed to demonstrate whether structural changes observed in the FORWARD trial are translated into a symptomatic benefit (Latourte et al. 2020).

Among the abovementioned approaches, Sprifermin™ is currently considered the only DMOAD promoting articular cartilage regeneration. The Wnt-inhibitor Lorecivivint® has also been intensively studied as a promising therapeutic candidate against OA, but clinical studies are still ongoing. Overall, the long-term effects of these and other potential DMOADs remain to be evaluated. The investigators report differential outcomes within the patient cohort, implying a subgroup-dependent responsiveness to Sprifermin™ and Lorecivivint®, respectively. In the section “Phenotypes, endotypes, and theratypes in OA”, we will take a closer look at different groups of OA patients and so-called “theratypes” (groups of patients who respond to a particular therapy).

Despite some rare successful reports, most preclinical studies testing potential DMOADs in animal OA models show strong results but fail in clinical trials. Many reasons explaining the discrepancies observed in pre-clinical and clinical studies are conceivable, and the most important ones will be discussed in the next section.

## Advantages and flaws of animal models for DMOAD testing

Despite the discrepancies regarding the efficacy of DMOADs in animal and clinical studies, the use of animal OA models remains essential. The key objective of animal models is to reproduce the entire pathogenesis of human OA by ideally taking different disease features into account, such as the involvement of various tissues, the multifactorial aetiology, the heterogeneity, as well as the monitorability and measurability of OA symptoms (Lorenz and Grassel 2014; Szponder et al. 2022; Longo et al. 2023). However, precisely because of the multifactorial aetiology and heterogeneity, it is unrealistic to generate results that allow conclusions with universal validity regarding potential treatment options by using only one model. This makes the choice of a suitable model quite challenging. Over the past decades, numerous animal models, comprising different species and models of OA (e.g., chemically or surgically induced OA), have been developed to study OA, which were already described in detail in previous articles (McCoy 2015; Szponder et al. 2022; Longo et al. 2023). Therefore, instead of repeating previously summarized information, the following subsections will focus on limitations and possible disadvantages of animal models used in preclinical studies regarding DMOAD treatments.

### Most frequently used animal models of OA

The different OA models include naturally occurring/age-dependent OA, PTOA induced by non-invasive or surgical techniques, chemically induced OA, and OA in genetically modified models (Lorenz and Grassel 2014; Longo et al. 2023). The most commonly used in vivo model is PTOA, even though only 12% of OA cases are caused by prior trauma (Hart 2022). Moreover, the most frequently used techniques to induce PTOA involve specific forms of surgical joint destabilization, such as destabilization of the medial meniscus (DMM), anterior cruciate ligament transection (ACLT), partial or total medial meniscectomy ((p)Mx or (p)MMx), or a combination of both (Mohan et al. 2011; Christiansen et al. 2015). In particular, the murine DMM model is considered highly reproducible. However, due to the mild OA progression, DMM is not always preferred for studies analyzing potential therapeutic interventions (McNulty et al. 2012). Moreover, male mice are predominantly used for the DMM model because female mice are less prone to developing OA (discussed below). In contrast to surgically induced experimental OA, the advantage of non-invasive techniques that induce cartilage damage (e.g., tibial compression overload) is that they mimic the pathogenesis of human OA more accurately (Christiansen et al. 2012, 2015; Rai et al. 2017). Chemical models of OA, such as the monoiodoacetate (MIA)-induced or collagenase-induced osteoarthritis model (CIOA), are also less invasive than surgical models and well reproducible. However, cartilage degradation in these models is one-sided and markedly faster than human OA progression (Longo et al. 2023). After all, the most natural OA pathogenesis can be observed in ageing animals spontaneously, which is unfortunately not solely time-consuming but also cost-intensive.

In addition, although OA has been described as a disease of the whole joint (Loeser et al. 2012), many studies focus only on one or two selected joint tissues during experimental OA progression. Only pathophysiological changes in articular cartilage are often considered, without considering the adjacent synovium or subchondral bone.

### Relevance of species, animal size, age, and sex

The most frequently used animals for OA models are rodents such as mice, guinea pigs, rats, and rabbits. These small animals reach skeletal maturity faster, as a prerequisite for OA induction, have a relatively rapid disease progression, purchase and housing are cheaper, and are easier to handle than larger animals like sheep, dogs, or horses (McCoy 2015; Serra and Soler 2019). The most commonly used small animals are mice due to the numerous available genetically engineered models and analytical tools (Gurumurthy and Lloyd 2019). However, it must be kept in mind that species-dependent differences in anatomy, joint biomechanics, and biochemical processes can lead to discrepant results when testing DMOADs (McCoy 2015). For example, the overall cartilage thickness in mice is about 50-fold thinner than in humans, calcified cartilage is thicker, and the zonal organization is different. In comparison, larger animals such as sheep and pigs are more similar to human joint anatomy (McCoy 2015). Also, species-associated biochemical differences have to be considered, such as the fact that in mice, transforming growth factor β3 (TGF-β3) is responsible for the activation of the respective receptors, while in humans, TGFβ signaling is mainly mediated by TGF-β1 (Poulsen et al. 2023). Furthermore, ADAMTS-5 is the primary aggrecanase responsible for aggrecan degradation in murine models (Glasson et al. 2005; Stanton et al. 2005), while ADAMTS-4 does not seem to play a relevant role (Glasson et al. 2004; Majumdar et al. 2007). In contrast, both aggrecanases are involved in matrix degradation in human OA (Song et al. 2007). Even the mouse strain can make a difference in murine models, as reported for canonical Wnt signaling in injury-induced and STR/ort mouse models (Jenei-Lanzl and Pongratz 2022; Longo et al. 2023). While Wnt signaling is upregulated in early OA in the synovium and cartilage in murine PTOA models, this did not occur in the STR/ort mouse (Poulsen et al. 2023).

An additional factor that influences outcomes is age. One must consider that OA is usually induced in young animals right after they reach skeletal maturity. This discrepancy with human OA pathogenesis can make a massive difference in searching for DMOADs. Accordingly, cartilage degradation and subchondral bone erosion were more severe in old mice than in younger animals (Huang et al. 2017). Moreover, OA-associated hyperalgesia is more severe and lasts longer in older rats than in young animals (Ro et al. 2020).

Although it is speculated that mainly peri- and postmenopausal women are affected by OA (Hanna et al. 2004; Hunter and Bierma-Zeinstra 2019), gender aspects are often ignored (Lorenz and Grassel 2014; Jenei-Lanzl and Pongratz 2022). However, male mice and rats are preferred for animal experiments, especially in PTOA models, because females are less prone to developing PTOA. One reason for sex-specific differences might be that cartilage thickness is significantly lower in females than males (Li et al. 2023b). Sex-dependent differences become particularly obvious when looking at OA-associated pain. Pain perception generally differs between women and men, where high estrogen and low testosterone levels are associated with increased pain levels (Dance 2019). Accordingly, significantly worse pre- and post-surgery knee pain was reported among women as compared to men (Perruccio et al. 2017, 2019, 2020; Liao et al. 2022), and a significantly lower central sensitization to pain was observed in men (Speed et al. 2017). Moreover, complement activation in female mice of commonly used mouse strains, particularly BALB/cJ, is limited by decreased expression of complement components associated with the terminal pathway (Kotimaa et al. 2016). This might result in a sex-specific protection against age- and injury-related OA development (Joos et al. 2015; Riegger et al. 2020, 2023a; Ruths et al. 2024).

### The issue of pain evaluation in animal models of OA

Since pain ranks as the cardinal symptom of OA, it is essential to include methods for monitoring the genesis and progression of joint pain in animal studies (Dekker et al. 1992). Unfortunately, only a few studies, dealing mainly with rodent models of OA, investigated the pain aspect(Vincent 2019).

One reason for the lack of consistent OA pain studies is, as already described above in detail, that existing rodent models replicate only specific OA phenotypes or even only mimic distinct OA stages. Therefore, the genesis and manifestation of joint pain in these models vary (Alves-Simoes 2022). The chemically induced MIA model of OA was the first to be used to investigate OA-related pain behavior. This model induces a relatively rapid and robust pain phenotype with strong inflammation, eventually resulting in degenerative changes. In contrast, surgical models instead produce joint degradation associated with inflammatory processes. Accordingly, paindevelops much more slowly (Yu et al. 2022).

Another reason is the limited availability of adequate pain measurement techniques. Classical behavioral tests to assess OA pain include measuring the mechanical withdrawal threshold (allodynia) using a von Frey filament or thermal hyperalgesia on the hot/cold plate (Alves-Simoes 2022). However, these tests require an external painful stimulus and are experimenter-dependent; therefore, their relevance is questionable (Ritter et al. 2023). More reliable tools have been developed in recent years, such as the static and dynamic weight-bearing systems that measure weight distribution asymmetry as a pain response or gait analysis. In particular, the latter can monitor OA-induced changes in locomotion by multidimensional analysis of temporal and spatial dynamics of gait (Ritter et al. 2023). Objective pain analysis in freely moving animals can also be performed using novel methods such as optogenetics and machine learning or in vivo imaging (Alves-Simoes 2022). However, standardization is required for these techniques as a prerequisite for the comparability of future studies.

### Lack of comorbidity models

Multi-morbidity of OA, or in other words, the coexistence of additional chronic conditions, is very common among OA patients. As published recently, 60% of patients suffering from OA are also affected by at least one comorbidity (Watt and Wise 2021). Cardiovascular diseases such as hypertension, metabolic diseases including diabetes and obesity, but also psychological disorders like depression, were identified as the most frequent comorbidities in OA (Calders and Van Ginckel 2018; Sohn and Jenei-Lanzl 2023). These comorbidities and OA often share risk factors and symptoms; thus, disease-modifying drugs might be helpful for more than one condition. For example, since most OA comorbidities are also accompanied by mild chronic inflammation, anti-inflammatory DMOADs might reduce the symptoms originating from all disorders. However, caution should be taken when choosing medication because, conversely, potential side effects may worsen OA or comorbidity symptoms, as previously described in the case of DMOAD toxicity, which might lead to gastrointestinal or heart problems (Watt and Wise 2021; Sohn and Jenei-Lanzl 2023).

Unfortunately, very few studies worked with OA comorbidity models in animals until now. For example, it has been demonstrated that OA progression is accelerated in obese or obese diabetic mice (Mooney et al. 2011; Lopez et al. 2023). In another study, higher synovitis scores and increased subchondral bone calcification were reported in hypertensive rats (Yeater et al. 2023). More recently, Julovi and colleagues established a novel chronic kidney disease (CKD) and OA co-morbidity mouse model and observed a significant bidirectional disease-promoting interaction between CKD and OA (Julovi et al. 2023). These examples do not only underline the relevance of OA phenotypes but also indicate that future studies should address comorbidity effects.

The above-described discrepancies between the preclinical animal models and clinical situation (summarized in Table 3) are a comprehensible reason for the disappointing outcomes of potential DMOADs in clinical trials and reveal the general challenges of translational research focusing on drug development. There is no gold standard for choosing one of the numerous OA animal models, because current models never fully mimic the human disease, as stated in several review articles (Yu et al. 2022; Alves-Simoes 2022).

**Table 3:** Main discrepancies between animal OA models and clinical situation

	**Animal studies (mainly mice)**	**Clinical situation**
Anatomical/structural differences	50-fold thinner cartilage, Pronounced calcified cartilage Low mechanical loading (quadrupedal, usage of tail)	Thickest knee articular cartilage of land mammals Pronounced intermediate/deep zones High mechanical loading (bipedal)
Type of OA	Injury-induced (PTOA), chemically-induced, rarely age-/obesity-related; very few studies on age-related OA (e.g., spontaneous/age-dependent OA, STR/ort or SAMP8 mice)	Mainly unclear/multifactorial cause, e.g., age, sex, obesity, comorbidities (see below); Only 12% of PTOA cases
Mechanistic differences	TGF-β3-mediated TGFβ signaling ADAMTS5: Primary aggrecanase ADAMTS4: No significant role in OA	TGF-β1-mediated TGF-β signaling Both ADAMTS4/5 are involved in OA pathogenesis
Sex-related differences	Male mice/rats used (more prone to/more reproducibly develop PTOA after surgery) Different concentrations of complement components in female mice/differences between strains	Higher prevalence of OA in women; worse pre- and post-surgery knee pain in women No sex-related differences in concentrations of complement components reported
Measurement of pain	Limited adequate pain measurement techniques/“silent suffering”	Subjective pain perception (e.g., WOMAC subscale); intake of analgesics over many years
Comorbidity in OA	Limited availability of animal co-morbidity OA models	60% of OA patients are affected by at least one comorbidity

### Alternative models to study DMOADs in OA

Scientists worldwide aim to reduce animal testing according to the 3Rs (replace, reduce, refine) (Little et al. 2022). In addition to the possibility of using lower organisms such as non-mammalian animals (e.g. zebrafish (Manivong et al. 2023)), various other alternatives have been developed or initiated. For example, 3-dimensional tissue-engineered organoids might resemble joint tissue structures. The most frequent organoid cultures are formed by chondrogenic progenitor cells obtained from the bone marrow or adipose tissue (Zeng et al. 2023). In addition, cartilage, bone or even osteochondral organoids can also be engineered using induced pluripotent stem cells, mature primary chondrocytes or bone cells (O’Connor et al. 2021; Abe et al. 2023).

Microfluidic organ-on-a-chip models may help to recapitulate the joint tissue environment and cell–cell interactions (Rothbauer et al. 2022). In particular when considering inflammation, a “synovial-joint-on-a-chip” using human tissue/cell sources (Banh et al. 2022; Li et al. 2023c) might be suitable to test the efficacy of novel DMOADs. However, the pain component is not yet integrated into these platforms. Using dorsal root ganglia in a microfluidic chip system might offer the solution to this dilemma but this technology is not yet fully developed (Moreau et al. 2024).

Finally, based on existing cell culture, animal study, or clinical trial data, AI models could be used to explore tissue interactions, to model biological processes or even to predict the efficacy of DMOADs (Foster et al. 2023).

Nevertheless, the lack of ideal models that accurately phenocopy human OA endotype is only one reason why might DMOADs fail in clinical settings. In the next chapter, we will discuss the broad heterogeneity in disease patterns to illustrate the enormous diversity among OA patients.

## Phenotypes, endotypes, and theratypes in OA

In the following sub-sections, we will provide insight into current concepts for the stratification of OA patients. In this context, the used terms are defined as follows: phenotype (grouping of OA patients according to clinical characteristics of the disease, excluding biochemical, physiological, and molecular properties), endotype (grouping of OA patients according to distinct features of mechanistic pathways and underlying mechanisms primarily driving disease progression), and theratype (grouping of OA patients according to their response to a certain set of therapeutics).

### The need to identify OA patient subgroups

As mentioned, OA is a multifactorial disease resulting in heterogeneous patient groups. Nevertheless, most previous studies on DMOAD development did not consider this aspect, which could be one of the significant reasons why respective clinical studies often fail. Therefore, stratification according to well-defined disease subtypes is a central point for improving the success rate of future OA studies (Mobasheri and Loeser 2024) and encouraging examples from chronic autoimmune diseases (e.g., severe asthma, rheumatoid arthritis, systemic lupus erythematosus) (Lewis et al. 2019, Schoettler and Strek 2020, Fasano et al. 2023, Cheng et al. 2024) fuel this hope as further described below.

During the last decade, numerous publications have addressed this topic. However, defining OA phenotypes and endotypes was a difficult task. By definition, a phenotype refers to a composite of observable characteristics or traits of an individual that result from genetic and environmental factors. In contrast, an endotype refers to a subtype of disease defined functionally and pathogenetically by a molecular mechanism or a treatment response (Anderson 2008; Deveza and Loeser 2018). Nevertheless, this general definition has not consistently been applied in the literature. In 2020, a panel of experts published the following definition in their framework for conducting and reporting OA phenotype research: „OA phenotypes are subtypes of OA that share distinct underlying pathobiological and pain mechanisms and their structural and functional consequences “ (van Spil et al. 2020). However, it should be remembered that different OA endotypes can lead to similar phenotypes, and an OA phenotype can result from different endotypes. Therefore, in the context of DMOAD development, a stratification according to the endotype should be of central importance to test the most effective drug in the most suitable patient cohort, or more precisely, to choose the right patient cohort matching the drug’s mode of action. In other words, there is an urgent need to identify different OA theratypes instead of still expecting the development of a one-size-fits-all panacea (Mobasheri and Loeser 2024). Nevertheless, phenotypic features related to disease progression or pain are also relevant to define more homogeneous treatment and control groups. In this context, an increased focus on pain as a patient-reported outcome measure (PROM) of illness has been recently claimed as a conclusion of the OARSI 2022 clinical trials symposium (Karsdal et al. 2023).

### Recent attempts to define OA phenotypes

First, experimental and clinical data have been used to define and characterize OA phenotypes from symptomatic and structural perspectives (Deveza and Loeser 2018). These phenotypes were categorized as mechanistic, prognostic, and response to therapy subgroups (Deveza and Loeser 2018; Dorio and Deveza 2022). OA phenotyping was mainly based on previous knowledge, unbiased cluster analysis of selected parameters, or completely unbiased omics studies of various biological sources. Thus, phenotyping approaches followed two major principles: Top-down phenotyping, which is hypothesis-driven, vs. step-up phenotyping without prior hypothesis to find clusters with very similar characteristics (Berenbaum 2019). Respective studies were mainly based on clinical characteristics, imaging data (specific structural features, e.g., from radiologic or MRI analysis), or biochemical parameters (e.g., biomarkers, cytokine profiles).

So far, a metabolic syndrome phenotype, an inflammatory phenotype, a bone and/or cartilage phenotype, and a mechanical overload/injury phenotype have been most frequently described in the literature. Furthermore, minimal joint disease (Knoop et al. 2011; Dell’Isola et al. 2016; Gijon-Nogueron et al. 2025), hormonal-driven/menopause (Karsdal et al. 2014; Gijon-Nogueron et al. 2025), genetically-driven (Karsdal et al. 2014), and ageing-driven phenotype (Mobasheri et al. 2017, 2019), as well as chronic pain or pain subtype phenotypes (Kittelson et al. 2016; Dell’Isola et al. 2016; Gijon-Nogueron et al. 2025), and muscle-/obesity-/depression-/psychological distress associated phenotypes (Knoop et al. 2011; Gijon-Nogueron et al. 2025), and a severe radiographic phenotype (Gijon-Nogueron et al. 2025) were suggested as classifiers.

As an example, a previous review included 24 studies and described six potential clinical phenotypes (chronic pain with central sensitization, inflammatory, metabolic syndrome, bone and cartilage metabolism, mechanical overload, and minimal joint disease as an outcome-based phenotype) (Dell’Isola et al. 2016). In a subsequent study, the same group tested this subgroup classification in a different knee OA cohort. Patients who could be assigned to more than one phenotype were classified as „complex knee OA “. 84% of the patients could be classified, with an overlap of 20% representing a proof of principle for this classification approach. The duration of the minimal joint disease phenotype was shorter, while the chronic pain phenotype included more women (Dell’Isola and Steultjens 2018).

There is growing evidence that metabolism is implicated in the pathogenesis of osteoarthritis (Mobasheri et al. 2017). Female patients from the Rotterdam Study with increased metabolic syndrome severity showed progression of osteophytes, bone marrow lesions, and cartilage defects, indicating more structural OA progression after five years (Jansen et al. 2023).

OA patients are mostly of advanced age and frequently have comorbidities, which might also influence the progression of OA. Therefore, the respective association was analyzed in the UK Clinical Practice Datalink GOLD database, and five clusters were identified: relatively healthy, cardiovascular, musculoskeletal-mental health, cardiovascular-musculoskeletal (CV-MSK), and metabolic (Swain et al. 2022). The CV-MSK cluster had the highest health care utilization and mortality risk. However, a possible association with OA-related clinical and structural features was not included and deserves further investigation.

Besides functional impairment, pain is a leading symptom of OA but does not necessarily correlate with structural damage of the affected joint. Therefore, pain phenotyping has gained more attentionand has been reviewed recently (Saxer et al. 2024).

Since women are not only more frequently affected by knee OA, but also exhibit differences regarding pain perception, concentrations of systemic inflammation markers, and structural cartilage features (Perruccio et al. 2017, 2019; Segal et al. 2024) – main outcome parameters in clinical trials – a sex-specific evaluation might be reasonable. It should be included in the study design.

### OA endotypes and biomarkers

For clinical trials on specific pharmacological interventions, classification according to endotypes seems to be most relevant. These approaches can be based on biomarker-defined phenotypes, single-omics (genomics, proteomics, metabolomics, etc.), or multi-omics studies (Mobasheri et al. 2021; Rai et al. 2023).

The concept of using biomarker profiling to define molecular endotypes of OA has been reviewed elsewhere (Mobasheri et al. 2019; Hannani et al. 2024). One study focusing on a single biomarker reported a low repair-driven endotype, which was identified based on systemic levels of the collagen type II propeptide (PRO-C2) as a marker of its biosynthesis and was associated with radiographic progression and superior response to the pro-anabolic drug salmon calcitonin (Luo et al. 2021). Interestingly, a subsequent Sprifermin™-subtype-study based on stratification by PRO-C2 revealed a structural effect which had not been detected without stratification (Bay-Jensen et al. 2022). An example for a biomarker panel-based approach has been published by Angelini et al.: In this study, 15 biomarkers of joint tissue turnover were determined in serum and urine, which allowed the identification of three dominant OA endotypes: low tissue turnover (low repair and articular cartilage/subchondral bone formation/resorption, cartilage degradation) – structural damage (high bone formation/resorption, cartilage degradation) – systemic inflammation (joint tissue degradation, inflammation, cartilage degradation) (Angelini et al. 2022). The low tissue turnover endotype included the highest proportion of non-progressors, the structural damage endotype was mainly linked to longitudinal structural progression, and the systemic inflammation endotype was linked to sustained or progressive pain.

Recently, interesting results from the phase III clinical trial testing JTA-004, a potential DMOAD composed of HA, plasma proteins, and clonidine, in knee OA patients have been reported. In this study, post-hoc analysis revealed that JTA-004 efficiently reduced symptoms of knee OA patients assigned to the endotype of synovial-driven OA, characterized by high levels of systemic inflammation (Henrotin et al. 2024). Thus, this report confirms the promising potential of phenotyping in terms of DMOAD development.

Taken together, the above-described approaches to classify different phenotypes and endotypes of OA patients seem to allow for the pre-selection of potentially “suitable”, or rather, “responding” patients (theratypes) for a particular class of DMOADs. Despite the notion of plausible phenotypes or endotypes, it remains challenging to assign a patient to a matching group precisely. This issue will be further addressed in Chapter 6, in which we will outline current personalized medicine strategies. But before, the third potential reason why DMOADs fail in clinical settings, namely the poor evaluability of the outcome, will be discussed.

## Lack of suitable clinical outcome parameters

As described above, most of the recently published clinical trials report disappointing outcomes, illustrating that the medications under investigation have either provided only short-term pain relief or shown no efficacy at all (Bennell et al. 2021; Migliorini et al. 2021; Rodriguez-Merchan 2023). This might also be related to the fact that a variety of tissues and signaling pathways are involved in the etiology of OA. As a result, this complicates the identification of specific targets and therapeutic approaches to prevent or improve OA-associated symptoms. It has to be noted that there is evidence that certain orthobiologics (e.g. platelet rich plasma (PrP) or HA) lead to an anti-inflammatory signaling with consecutive pain relieve in OA-patients ^(^^Zhao et al.2020^; ^Szwedowski et al.2021^^). However, the i.a. administration of these substances is controversially discussed among clinicians. A reason for this is the complex process of clinical outcome evaluation.^

### Outcome evaluation: objective measurement of subjective parameters

A significant challenge complicating a profound qualitative analysis of different treatment options is the presence of distinct endotype-related signaling clusters, all leading to the common final pathway of OA. In daily clinical practice, identifying the exact etiology of OA is often tricky, especially in cases with severe joint degeneration. Furthermore, different clusters might overlap in a single patient (e.g., structural damage due to posttraumatic cartilage lesions and joint inflammation due to concurrent joint instability).

Another problem regarding the evaluation of outcome parameters is based on the fact that, in contrast to animal models, no histopathologic examination can be undertaken. Consequently, radiologic assessment (e.g., MRI) or subjective evaluations such as PROMs are surrogate parameters in clinical studies. Given that up to 60% of adult patients with radiographic evidence of knee OA are asymptomatic, the significance of PROMs as a key indicator of DMOAD-related clinical improvements cannot be overstated (Dillon et al. 2006).

To date, there are several PROM evaluation tools regarding osteoarthritis. The most frequently used scores are the Medical Outcome Studies Short Form 36 (SF-36) and EUROQoL (EQ5-D) questionnaires (Lundgren-Nilsson et al. 2018). Newer evaluation tools, such as the Subjective Hip Value and Subjective Knee Value, represent single-item surveys. Despite – or potentially because of – the simple structure of these surveys, the latter serve as both responsive and reliable instruments for assessing joint function (Krueger et al. 2020; Plachel et al. 2022; Leopold et al. 2024).

Nevertheless, PROMs are additionally influenced by mental health, individual expectations, and lifestyle modifications (Kraus et al. 2021; Goh et al. 2021; Orr et al. 2022). This highlights the inherent complexity regarding the individual weighting of different parameters and their limitations in objectively measuring respective outcome parameters. Although the patients’ subjective perception might highly influence PROMs, personal satisfaction is eventually the primary aim of a treatment.

### Clinical outcome analysis of subjective and objective criteria

General performance and pain surveys can be distinguished from joint−specific measures such as the WOMAC, Oxford Knee Score (OKS), or Knee Injury and Osteoarthritis Outcome Score (KOOS). From a clinical perspective, these specific questionnaires are valid instruments through a combined evaluation of objective (functional evaluation) and subjective (pain assessment) outcome parameters (Collins et al. 2011). These questionnaires represent the gold standard for assessing therapeutic approaches in patients with joint−related diseases and their impact on daily living and quality of life. In 2025, there will be a variety of joint−dependent evaluation scores. By combining objective and subjective measurements, most of these items can be considered as reliable, valid, and responsive instruments in evaluating OA−related joint pathologies and corresponding treatment strategies (Gandek and Ware 2017; Zhang et al. 2023).

Overall, clinicians and researchers are well aware of the weaknesses of the outcome parameters most commonly used for standardized description of clinical trials. Although the classical parameters might remain unchanged, additional criteria will likely complement and improve the evaluation procedure in the future. In the next section, a current hot topic, which might substantially enhance drug development in general and the context of OA, will be addressed: the utilization of big data in personalized medicine.

## Advances in personalized medicine in OA

In principle, the high failure rate of DMOADs in clinical trials does not seem to be a disease-specific issue per se. Approximately 90% of drugs are estimated to fail during clinical trials and will never obtain approval, as reviewed elsewhere (Sun et al. 2022). Especially in diseases characterized by a high heterogeneity among the patients, such as cancer, personalized or precision medicine has emerged as a promising strategy to increase therapeutic success in recent years. By applying novel -omics technologies, it is now possible to identify suitable therapeutic approaches for individual patients or specific subgroups (Gambardella et al. 2020). In the following section, we will examine the development of drugs in another incurable arthritic joint disease, RA. We will discuss the novel approaches used to increase the success rate in clinical trials. Further, we will outline how -omics are currently being used to optimize the stratification of phenotypes and endotypes in OA.

### Lessons learned from disease-modifying antirheumatic drugs – a success story

RA is an autoimmune disorder affecting joints and is characterized by strong synovitis and severe pain occurring in recurrent flares. In contrast to OA, several disease-modifying antirheumatic drugs (DMARDs) have proven effective in clinical trials. DMARDs are classified into conventional synthetic (e.g., methotrexate) and biological drugs, which target a specific mediator, cell type, or pathway of the immune system (e.g., anti-TNF drugs, T cell co-stimulation inhibitors, IL-6 receptor inhibitors, and Janus kinase inhibitors). In particular, the combination of methotrexate, which mainly promotes anti-inflammatory effects by increasing adenosine levels, with a biological drug appears to be a practical approach to mitigate disease activity and joint degeneration (Hazlewood et al. 2016). However, not all RA patients respond equally to any given therapeutic strategy. Therefore, the treatment of RA patients with DMARDs has been mainly based on a “trial-and-error” approach, resulting in a limited efficacy in about 40% of the cases, the so-called non-responders or difficult-to-treat patients (Buch 2018). Concerning the striking heterogeneity in RA patients, which rules out the one‐drug‐fits‐all concept, researchers and clinicians agree that a fundamental shift towards precision medicine is needed (Wang et al. 2021).

Using transcriptomic data and multi-omics, drug-specific transcription and/or DNA methylome signatures for the anti-TNF response have been identified in RA synovium and blood-derived immune cells from affected patients, allowing a prediction of the response to anti-TNF drugs in RA therapy (Aterido et al. 2019; Tao et al. 2021). By incorporating machine learning using the molecular markers identified by the omics approaches, reliable models were developed to predict individual patients’ expected response before anti-TNF treatment (Tao et al. 2021).

Multi-omics can also be applied at the single cell level, resulting in a higher resolution enabling the discrimination of cell type-specific differences and allowing a more precise classification of RA patients into subgroups (Wang et al. 2021). In this context, single-cell technology, for example, revealed individualized repertoires of B cell receptors in RA patients originating from differential antigen exposure during life, including receptors specific for cyclic citrullinated peptides and other RA-associated autoantigens (Xu et al. 2019). This knowledge can contribute to designing tailored antigen-targeted therapeutics and support the individual selection of suitable drugs for patients.

Bioinformatics and biostatistics are essential to effectively utilizing the vast potential of omics-derived data. Using sophisticated methods, including artificial intelligence, deep learning, and machine learning, it is possible to establish future models to predict RA progression or therapeutic outcomes. This topic has been recently reviewed elsewhere (Momtazmanesh et al. 2022).

In conclusion, combining multi-omics and bioinformatics is a groundbreaking approach to optimizing drug efficacy based on molecular endotypes. This accounts for cancer and RA and might also represent an attractive strategy in OA. Although individualized or tailored therapies are costly and time-consuming, considering genetic diversity, individual lifestyle, and environment during the selection of the patient cohort might help to reduce the high failure rate of DMOADs in clinical trials. Nevertheless, it should be noted that the effectiveness of DMARDs primarily relies on their anti-inflammatory effects and thus symptomatic relief. In contrast, DMOADs should address pain and inflammation and promote cartilage regeneration, which remains an unmet need.

### Omics and big data in pheno-/endotyping of OA patients

Besides single or combined biomarker approaches, high-throughput omics technologies combined with sophisticated big data analyses have been used in OA endotype studies (Soul et al. 2018; Boer et al. 2021). The methodological approaches may comprise genomics, transcriptomics, proteomics, metabolomics, lipidomics or glycomics and can be based on joint tissues, cells derived thereof, synovial fluid, serum/plasma or urine. These approaches have the potential to provide novel insights into the highly complex and heterogeneous pathogenetic basis of OA, to identify new druggable targets and to define novel endotypes (Mobasheri et al. 2021; Rai et al. 2023). By means of a large multicohort genome-wide association meta-analysis, 100 OA-associated genetic loci have been identified which were overlapping to a considerable extent with respect to the affected joint site. Furthermore, the identified loci were also partly different in weight-bearing and non-weight-bearing joints and were even unique for a specific OA localization in some cases (Boer et al. 2021). Furthermore, sophisticated computational tools were used in this study to analyze likely involved effector genes and biological pathways. These insights may help to predict potentially effective drugs for specific OA sub-populations as described in case of RA above.

Using cartilage transcriptome analysis, four clusters could be identified that may define OA endotypes. Cluster 1: Glycosaminoglycan metabolic disorder, cluster 2: Collagen metabolic disorder, cluster 3: Activated sensory neurons, and cluster 4: Inflammation. Comparison with clinical features indicated that most severe osteophytes were present in cluster 2 and most pronounced joint space narrowing in cluster 4 (Yuan et al. 2020).

A recent study indicated that synovial fibroblasts from hand, hip, knee, and foot joints are independently affected by obesity, joint loading, and anatomical site. Furthermore, in this study, single-cell RNA sequencing could define different molecular endotypes of synovial fibroblasts (Wijesinghe et al. 2023). A possible association of their relative amount with clinical and/or prognostic features might help patient stratification for clinical trials in the future. Based on a plasma metabolomic analysis, three endotypes were suggested: cluster A, muscle weakness (higher concentration of butyrylcarnitine – C4); Cluster B, arginine deficit (lower arginine concentration); and Cluster C, low inflammatory OA (lower concentration of lysophosphatidylcholine) (Werdyani et al. 2021). Cluster A had the highest body mass index (BMI) and prevalence of diabetes, Cluster B the highest prevalence of coronary heart disease, and Cluster C the highest prevalence of osteoporosis. Another study on the blood metabolome of OA patients demonstrated that senescence endotypes identified by transcriptomic analysis in OA cartilage overlap with metabolic clusters in blood (Boone et al. 2024). These examples indicate that omics studies on local or systemic biological samples from OA patients allow clustering of subgroups and may markedly increase the knowledge on endotypes. However, a significant and still unsolved question is whether such results relate to the pathogenetic basis or the current disease stage of osteoarthritis.

### Specify the time window of therapeutic opportunity in OA

A special interest in early-stage OA has emerged based on the assumption that progression of OA might only be arrested or delayed before an irreversible joint destruction occurs (Im 2022). A respective “window of opportunity” has also been described for RA therapy (Burgers et al. 2019). Reliable diagnosis of early-stage OA is complex, and several criteria have been suggested (Madry et al. 2016; Migliore et al. 2017; Mahmoudian et al. 2021b). As an example, classification criteria proposed in 2018 by an international consortium included the patient-based questionnaire KOOS (two out of four subscales ≤ 85%), presence of joint line tenderness and/or crepitations, and radiologic KL score of 0–1 in the affected joint (Luyten et al. 2018). The clinical relevance of these criteria to predict structural and clinical OA progression was subsequently tested in probands from the Osteoarthritis Initiative. A substantial enrichment compared to controls was confirmed after 48 and 96 months, which was further improved by including joint effusion and/or Heberden´s nodes in the set of criteria (Mahmoudian et al. 2021a). Alternatively, classification criteria for early-stage OA based on MRI or arthroscopic findings have been suggested (Mahmoudian et al. 2021b).

Overall, previous efforts in the development of DMOADs have been mainly unsuccessful. Thus, a new path needs to be taken to address the individual needs of OA patients. Personalized medicine has been a successful approach in RA and cancer but might also be suitable for other diseases characterized by heterogeneous endotypes, such as OA. The rapid advances in developing new, “intelligent” software for big data analysis will undoubtedly contribute to a more precise endotyping of OA patients. This might allow specification of patient groups (theratypes), which will potentially benefit from specific DMOAD treatments. Besides choosing the appropriate therapeutic approach for a particular group of OA patients, the optimal timing might significantly impact drug efficacy.

## Conclusions

Current developments regarding OA phenotypes and endotypes have confirmed that this chronic disease’s heterogeneity is tremendous, representing a major problem for clinical trials. Despite considerable progress in the field, a generally accepted and applicable phenotype or endotype classification has not yet been established. Because of the incredibly multifaceted nature of OA, such a goal seems somewhat unrealistic. Therefore, the focus of stratification on relevant endotype features related to the tested drug and phenotype features associated with the primary outcome parameters for approval (PROMs—including pain—and structural features) seems to represent an appropriate and achievable goal for DMOAD development in the near future.

Accordingly, preselection of suitable endotypes that most likely benefit from the DMOAD being tested, in other words, theratypes, should be considered in clinical trials. Moreover, a profound post-hoc analysis of concluded trials, which did not achieve the chosen endpoints at first sight, might be worthwhile. It might be possible to identify differentially responding subpopulations as reported in the cases of the Wnt inhibitor Lorecivivint® or Sprifermin™.

Another critical aspect of clinical trial planning, which we have discussed above, is the rethinking of suitable and endotype-specific outcome parameters. Overall, it might be possible that the therapeutic effects of some of the past “ineffective” OA treatment strategies or DMOADs have been missed. In this context, repurposing drugs that failed in previous studies or were initially developed for other diseases or even OA comorbidities might have a high potential to treat specific OA subgroups effectively. Given the association between OA and other diseases, in particular metabolic syndrome (e.g., type 2 diabetes) and cardiovascular diseases (e.g., hypertension, hypercholesterolemia), many OA patients already receive medication to treat the respective comorbidities. The beneficial effects of repurposed drugs and challenges and future strategies in clinical drug repurposing in OA patients were previously reviewed elsewhere (Oo and Hunter 2022; Kuswanto and Baker 2024). However, not only does the final step of drug development explain the failure of DMOADs, but upstream testing conducted in in vitro or animal models also has obvious weaknesses. More complex in vitro models, such as organoids or a joint-on-a-chip, and new in vivo models, which better mimic the clinical picture of human? OA, including comorbidities, aging, and sex differences, needs to be considered.

**Fig. 8:**
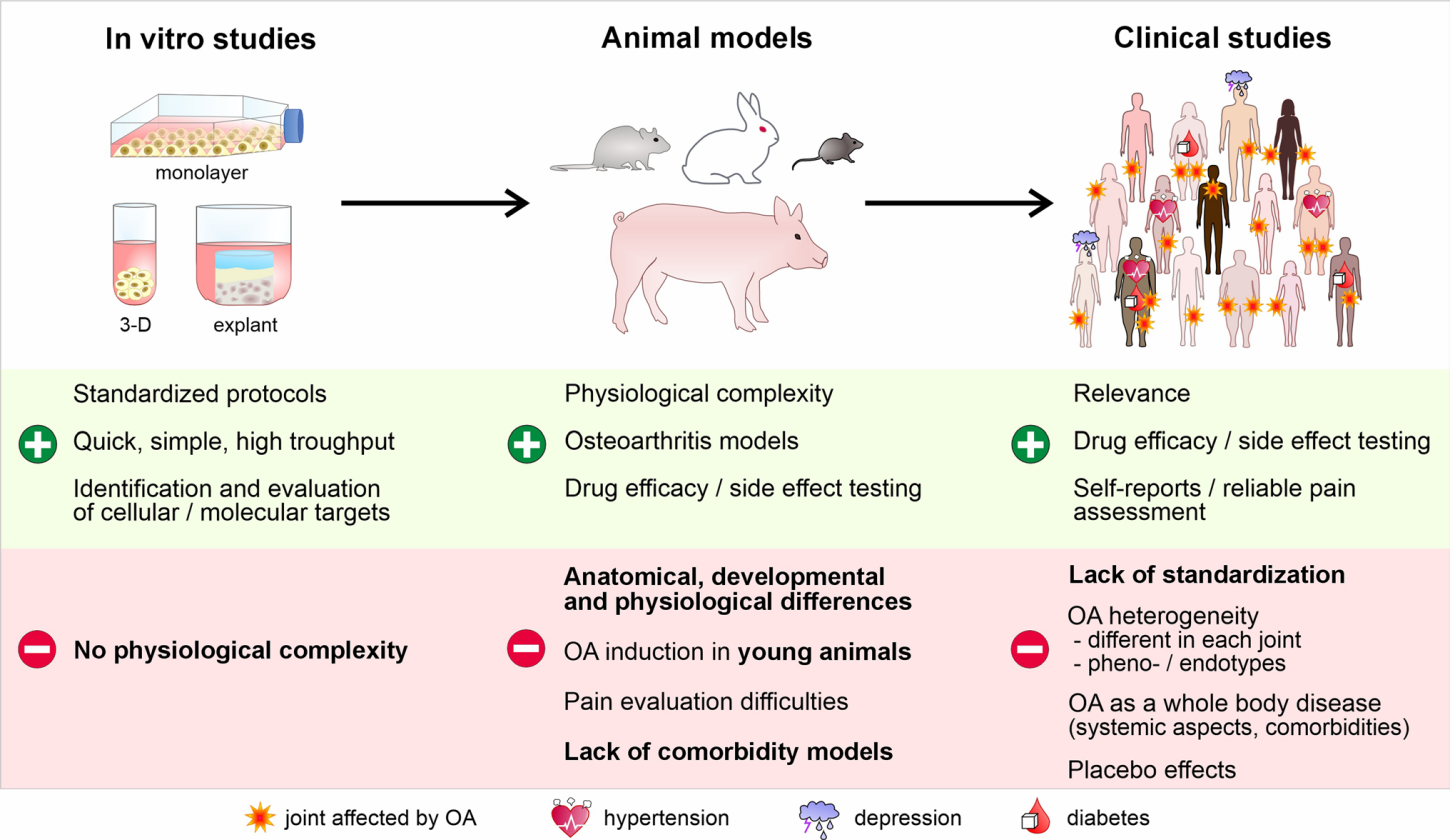
Real-life conditions of drug development. Although the theoretical procedure of drug testing – from in vitro studies, over animal models, and finally to application in clinical trials – will still prove reasonable in the future, it is essential to recognize the specific strengths and weaknesses of each step, as they may lead to potential discrepancies in the outcome

Altogether, a refinement of the current drug development procedure is needed, but has already been initiated by understanding and heeding the strengths and weaknesses of the single steps (Fig. 8).

## References

[CR1] Abe K, Yamashita A, Morioka M, Horike N, Takei Y, Koyamatsu S, Okita K, Matsuda S, Tsumaki N (2023) Engraftment of allogeneic iPS cell-derived cartilage organoid in a primate model of articular cartilage defect. Nat Commun 14(1):804. 10.1038/s41467-023-36408-0 36808132 10.1038/s41467-023-36408-0 PMC9941131

[CR2] Alves-Simoes M (2022) Rodent models of knee osteoarthritis for pain research. Osteoarthr Cartil 30(6):802–814. 10.1016/j.joca.2022.01.010 10.1016/j.joca.2022.01.010 35139423

[CR3] Anderson GP (2008) Endotyping asthma: new insights into key pathogenic mechanisms in a complex, heterogeneous disease. Lancet 372(9643):1107–1119. 10.1016/S0140-6736(08)61452-X 18805339 10.1016/S0140-6736(08)61452-X

[CR4] Angelini F, Widera P, Mobasheri A, Blair J, Struglics A, Uebelhoer M, Henrotin Y, Marijnissen AC, Kloppenburg M, Blanco FJ, Haugen IK, Berenbaum F, Ladel C, Larkin J, Bay-Jensen AC, Bacardit J (2022) Osteoarthritis endotype discovery via clustering of biochemical marker data. Ann Rheum Dis 81(5):666–675. 10.1136/annrheumdis-2021-221763 35246457 10.1136/annrheumdis-2021-221763

[CR5] Ashruf OS, Ansari MY (2022) Natural compounds: potential therapeutics for the inhibition of cartilage matrix degradation in osteoarthritis. Life 13(1):102. 10.3390/life13010102 36676051 10.3390/life13010102 PMC9866583

[CR6] Aterido A, Canete JD, Tornero J, Blanco F, Fernandez-Gutierrez B, Perez C, Alperi-Lopez M, Olive A, Corominas H, Martinez-Taboada V, Gonzalez I, Fernandez-Nebro A, Erra A, Lopez-Lasanta M, Lopez Corbeto M, Palau N, Marsal S, Julia A (2019) A combined transcriptomic and genomic analysis identifies a gene signature associated with the response to anti-TNF therapy in rheumatoid arthritis. Front Immunol. 10.3389/fimmu.2019.01459 31312201 10.3389/fimmu.2019.01459 PMC6614444

[CR7] Banh L, Cheung KK, Chan MWY, Young EWK, Viswanathan S (2022) Advances in organ-on-a-chip systems for modelling joint tissue and osteoarthritic diseases. Osteoarthr Cartil 30(8):1050–1061. 10.1016/j.joca.2022.03.012 10.1016/j.joca.2022.03.012 35460872

[CR8] Bay-Jensen AC, Manginelli AA, Karsdal M, Luo Y, He Y, Michaelis M, Guehring H, Ladel C (2022) Low levels of type II collagen formation (PRO-C2) are associated with response to sprifermin: a pre-defined, exploratory biomarker analysis from the FORWARD study. Osteoarthr Cartil 30(1):92–99. 10.1016/j.joca.2021.10.008 10.1016/j.joca.2021.10.008 34737064

[CR9] Bedingfield SK, Colazo JM, Yu F, Liu DD, Jackson MA, Himmel LE, Cho H, Crofford LJ, Hasty KA, Duvall CL (2021) Amelioration of post-traumatic osteoarthritis via nanoparticle depots delivering small interfering RNA to damaged cartilage. Nat Biomed Eng 5(9):1069–1083. 10.1038/s41551-021-00780-3 34413494 10.1038/s41551-021-00780-3 PMC8497446

[CR10] Bennell KL, Paterson KL, Metcalf BR, Duong V, Eyles J, Kasza J, Wang Y, Cicuttini F, Buchbinder R, Forbes A, Harris A, Yu SP, Connell D, Linklater J, Wang BH, Oo WM, Hunter DJ (2021) Effect of intra-articular platelet-rich plasma vs placebo injection on pain and medial tibial cartilage volume in patients with knee osteoarthritis: the RESTORE randomized clinical trial. JAMA 326(20):2021–2030. 10.1001/jama.2021.19415 34812863 10.1001/jama.2021.19415 PMC8611484

[CR11] Berenbaum F (2019) Deep phenotyping of osteoarthritis: a step forward. Ann Rheum Dis 78(1):3–5. 10.1136/annrheumdis-2018-213864 30154088 10.1136/annrheumdis-2018-213864

[CR12] Bihlet AR, Balchen T, Goteti K, Sonne J, Ladel C, Karsdal MA, Ona V, Moreau F, Waterhouse R, Bay-Jensen AC, Guehring H (2024) Safety, tolerability, and pharmacodynamics of the ADAMTS-5 nanobody M6495: two phase 1, single-center, double-blind, randomized, placebo-controlled studies in healthy subjects and patients with osteoarthritis. ACR Open Rheumatol 6(4):205–213. 10.1002/acr2.11610 38311369 10.1002/acr2.11610 PMC11016567

[CR13] Boer CG, Hatzikotoulas K, Southam L, Stefánsdóttir L, Zhang Y, de Almeida RC, Wu TT, Zheng J, Hartley A, Teder-Laving M, Skogholt AH (2021) Deciphering osteoarthritis genetics across 826690 individuals from 9 populations. Cell 184(18):4784–4818. 10.1016/j.cell.2021.07.038 34450027 10.1016/j.cell.2021.07.038 PMC8459317

[CR14] Boone I, Tuerlings M, Coutinho De Almeida R, Lehmann J, Ramos Y, Nelissen R, Slagboom E, De Keizer P, Meulenbelt I (2024) Identified senescence endotypes in aged cartilage are reflected in the blood metabolome. Geroscience 46(2):2359–2369. 10.1007/s11357-023-01001-2 37962736 10.1007/s11357-023-01001-2 PMC10828277

[CR15] Brebion F, Gosmini R, Deprez P, Varin M, Peixoto C, Alvey L, Jary H, Bienvenu N, Triballeau N, Blanque R, Cottereaux C, Christophe T, Vandervoort N, Mollat P, Touitou R, Leonard P, De Ceuninck F, Botez I, Monjardet A, Van Der Aar E, Amantini D (2021) Discovery of GLPG1972/S201086, a potent, selective, and orally bioavailable ADAMTS-5 inhibitor for the treatment of osteoarthritis. J Med Chem 64(6):2937–2952. 10.1021/acs.jmedchem.0c02008 33719441 10.1021/acs.jmedchem.0c02008

[CR16] Briat A, Jacques C, Malige M, Sudre L, Nourissat G, Auzeloux P, Guehring H, Cachin F, Berenbaum F, Miot-Noirault E (2022) (99m)Tc-NTP 15–5 is a companion radiotracer for assessing joint functional response to sprifermin (rhFGF-18) in a murine osteoarthritis model. Sci Rep 12(1):8146. 10.1038/s41598-022-11080-4 35581224 10.1038/s41598-022-11080-4 PMC9113995

[CR17] Buch MH (2018) Defining refractory rheumatoid arthritis. Ann Rheum Dis 77(7):966–969. 10.1136/annrheumdis-2017-212862 29588276 10.1136/annrheumdis-2017-212862

[CR18] Burgers LE, Raza K, Van Der Helm-Van Mil AH (2019) Window of opportunity in rheumatoid arthritis - definitions and supporting evidence: from old to new perspectives. RMD Open 5(1):e000870. 10.1136/rmdopen-2018-000870 31168406 10.1136/rmdopen-2018-000870 PMC6525606

[CR19] Calders P, Van Ginckel A (2018) Presence of comorbidities and prognosis of clinical symptoms in knee and/or hip osteoarthritis: a systematic review and meta-analysis. Semin Arthritis Rheum 47(6):805–813. 10.1016/j.semarthrit.2017.10.016 29157670 10.1016/j.semarthrit.2017.10.016

[CR20] Chaib S, Tchkonia T, Kirkland JL (2022) Cellular senescence and senolytics: the path to the clinic. Nat Med 28(8):1556–1568. 10.1038/s41591-022-01923-y 35953721 10.1038/s41591-022-01923-y PMC9599677

[CR21] Cheng J, Li M, Bai R (2022) The Wnt signaling cascade in the pathogenesis of osteoarthritis and related promising treatment strategies. Front Physiol 13:954454. 10.3389/fphys.2022.954454 36117702 10.3389/fphys.2022.954454 PMC9479192

[CR22] Cheng X, Meng X, Chen R, Song Z, Li S, Wei S, Lv H, Zhang S, Tang H, Jiang Y, Zhang R (2024) The molecular subtypes of autoimmune diseases. Comput Struct Biotechnol J 23:1348–1363. 10.1016/j.csbj.2024.03.026 38596313 10.1016/j.csbj.2024.03.026 PMC11001648

[CR23] Chevalier X, Giraudeau B, Conrozier T, Marliere J, Kiefer P, Goupille P (2005) Safety study of intraarticular injection of interleukin 1 receptor antagonist in patients with painful knee osteoarthritis: a multicenter study. J Rheumatol 32(7):1317–1323 15996071

[CR24] Chevalier X, Goupille P, Beaulieu AD, Burch FX, Bensen WG, Conrozier T, Loeuille D, Kivitz AJ, Silver D, Appleton BE (2009) Intraarticular injection of anakinra in osteoarthritis of the knee: a multicenter, randomized, double-blind, placebo-controlled study. Arthritis Rheum 61(3):344–352. 10.1002/art.24096 19248129 10.1002/art.24096

[CR25] Childs BG, Durik M, Baker DJ, Van Deursen JM (2015) Cellular senescence in aging and age-related disease: from mechanisms to therapy. Nat Med 21(12):1424–1435. 10.1038/nm.4000 26646499 10.1038/nm.4000 PMC4748967

[CR26] Chin AF, Han J, Clement CC, Choi Y, Zhang H, Browne M, Jeon OH, Elisseeff JH (2023) Senolytic treatment reduces oxidative protein stress in an aging male murine model of post-traumatic osteoarthritis. Aging Cell 22(11):e13979. 10.1111/acel.13979 37749958 10.1111/acel.13979 PMC10652304

[CR27] Chockalingam PS, Sun W, Rivera-Bermudez MA, Zeng W, Dufield DR, Larsson S, Lohmander LS, Flannery CR, Glasson SS, Georgiadis KE, Morris EA (2011) Elevated aggrecanase activity in a rat model of joint injury is attenuated by an aggrecanase specific inhibitor. Osteoarthr Cartil 19(3):315–323. 10.1016/j.joca.2010.12.004 10.1016/j.joca.2010.12.004 21163358

[CR28] Christiansen BA, Anderson MJ, Lee CA, Williams JC, Yik JH, Haudenschild DR (2012) Musculoskeletal changes following non-invasive knee injury using a novel mouse model of post-traumatic osteoarthritis. Osteoarthr Cartil 20(7):773–782. 10.1016/j.joca.2012.04.014 10.1016/j.joca.2012.04.014 22531459

[CR29] Christiansen BA, Guilak F, Lockwood KA, Olson SA, Pitsillides AA, Sandell LJ, Silva MJ, Van Der Meulen MC, Haudenschild DR (2015) Non-invasive mouse models of post-traumatic osteoarthritis. Osteoarthr Cartil 23(10):1627–1638. 10.1016/j.joca.2015.05.009 10.1016/j.joca.2015.05.009 PMC4577460 26003950

[CR30] Clement-Lacroix P, Little CB, Smith MM, Cottereaux C, Merciris D, Meurisse S, Mollat P, Touitou R, Brebion F, Gosmini R, De Ceuninck F, Botez I, Lepescheux L, Van Der Aar E, Christophe T, Vandervoort N, Blanque R, Comas D, Deprez P, Amantini D (2022) Pharmacological characterization of GLPG1972/S201086, a potent and selective small-molecule inhibitor of ADAMTS5. Osteoarthr Cartil 30(2):291–301. 10.1016/j.joca.2021.08.012 10.1016/j.joca.2021.08.012 34626798

[CR31] Coleman MC, Goetz JE, Brouillette MJ, Seol D, Willey MC, Petersen EB, Anderson HD, Hendrickson NR, Compton J, Khorsand B, Morris AS, Salem AK, Fredericks DC, Mckinley TO, Martin JA (2018) Targeting mitochondrial responses to intra-articular fracture to prevent posttraumatic osteoarthritis. Sci Transl Med. 10.1126/scitranslmed.aan5372 29437147 10.1126/scitranslmed.aan5372 PMC5987523

[CR32] Collaborators GBDO (2023) Global, regional, and national burden of osteoarthritis, 1990–2020 and projections to 2050: a systematic analysis for the Global Burden of Disease Study 2021. Lancet Rheumatol 5(9):e508–e522. 10.1016/S2665-9913(23)00163-7 37675071 10.1016/S2665-9913(23)00163-7 PMC10477960

[CR33] Collins NJ, Misra D, Felson DT, Crossley KM, Roos EM (2011) Measures of knee function: international knee documentation committee (IKDC) subjective knee evaluation form, knee injury and osteoarthritis outcome score (KOOS), knee injury and osteoarthritis outcome score physical function short form (KOOS-PS), knee outcome survey activities of daily living scale (KOS-ADL), lysholm knee scoring scale, oxford knee score (OKS), western ontario and mcmaster universities osteoarthritis index (WOMAC), activity rating scale (ARS), and tegner activity score (TAS). Arthr Care Res 63(11):208–228. 10.1002/acr.20632 10.1002/acr.20632 PMC4336550 22588746

[CR34] Conaghan P, Chevalier X, Mindeholm L, Schramm U, Praestgaard J, Seroutou A, Roubenoff R, Schieker M (2021) Intra-articular canakinumab (anti-interleukin-1β) for treatment of symptomatic knee osteoarthritis: a randomized, double-blind, placebo and naproxen-controlled phase II study: abstract number: 0728. Arthr Rheumatol 73:1474–1475

[CR35] Coryell PR, Diekman BO, Loeser RF (2021) Mechanisms and therapeutic implications of cellular senescence in osteoarthritis. Nat Rev Rheumatol 17(1):47–57. 10.1038/s41584-020-00533-7 33208917 10.1038/s41584-020-00533-7 PMC8035495

[CR36] Dance A (2019) Why the sexes don’t feel pain the same way. Nature 567(7749):448–450. 10.1038/d41586-019-00895-3 30918396 10.1038/d41586-019-00895-3

[CR37] Dekker J, Boot B, Van Der Woude LH, Bijlsma JW (1992) Pain and disability in osteoarthritis: a review of biobehavioral mechanisms. J Behav Med 15(2):189–214. 10.1007/BF00848325 1533877 10.1007/BF00848325

[CR38] Deligiannidou GE, Papadopoulos RE, Kontogiorgis C, Detsi A, Bezirtzoglou E, Constantinides T (2020) Unraveling natural products’ role in osteoarthritis management—An overview. Antioxid 9(4):348. 10.3390/antiox9040348 10.3390/antiox9040348 PMC7222394 32340224

[CR39] Dell’isola A, Steultjens M (2018) Classification of patients with knee osteoarthritis in clinical phenotypes: data from the osteoarthritis initiative. PLoS ONE 13(1):e0191045. 10.1371/journal.pone.0191045 29329325 10.1371/journal.pone.0191045 PMC5766143

[CR40] Dell’isola A, Allan R, Smith SL, Marreiros SS, Steultjens M (2016) Identification of clinical phenotypes in knee osteoarthritis: a systematic review of the literature. BMC Musculoskelet Disord 17(1):425. 10.1186/s12891-016-1286-2 27733199 10.1186/s12891-016-1286-2 PMC5062907

[CR41] Deshmukh V, Hu H, Barroga C, Bossard C, Kc S, Dellamary L, Stewart J, Chiu K, Ibanez M, Pedraza M, Seo T, Do L, Cho S, Cahiwat J, Tam B, Tambiah JRS, Hood J, Lane NE, Yazici Y (2018) A small-molecule inhibitor of the Wnt pathway (SM04690) as a potential disease modifying agent for the treatment of osteoarthritis of the knee. Osteoarthr Cartil 26(1):18–27. 10.1016/j.joca.2017.08.015 10.1016/j.joca.2017.08.015 28888902

[CR42] Deshmukh V, O’Green AL, Bossard C, Seo T, Lamangan L, Ibanez M, Ghias A, Lai C, Do L, Cho S, Cahiwat J (2019) Modulation of the Wnt pathway through inhibition of CLK2 and DYRK1A by lorecivivint as a novel, potentially disease-modifying approach for knee osteoarthritis treatment. Osteoarthr Cartil 27(9):1347–1360. 10.1016/j.joca.2019.05.006s 10.1016/j.joca.2019.05.006 31132406

[CR43] Deveza LA, Loeser RF (2018) Is osteoarthritis one disease or a collection of many? Rheumatology (Oxford). 10.1093/rheumatology/kex417 29267932 10.1093/rheumatology/kex417 PMC6251697

[CR44] Dillon CF, Rasch EK, Gu Q, Hirsch R (2006) Prevalence of knee osteoarthritis in the United States: arthritis data from the Third National Health and Nutrition Examination Survey 1991–94. J Rheumatol 33(11):2271–2279 17013996

[CR45] Dorio M, Deveza LA (2022) Phenotypes in osteoarthritis: why do we need them and where are we at? Clin Geriatr Med 38(2):273–286. 10.1016/j.cger.2021.11.002 35410680 10.1016/j.cger.2021.11.002

[CR46] Eckstein F, Wirth W, Guermazi A, Maschek S, Aydemir A (2015) Brief report: intraarticular sprifermin not only increases cartilage thickness, but also reduces cartilage loss: location-independent post hoc analysis using magnetic resonance imaging. Arthritis Rheumatol 67(11):2916–2922. 10.1002/art.39265 26138203 10.1002/art.39265 PMC5061102

[CR47] Eckstein F, Hochberg MC, Guehring H, Moreau F, Ona V, Bihlet AR, Byrjalsen I, Andersen JR, Daelken B, Guenther O, Ladel C, Michaelis M, Conaghan PG (2021) Long-term structural and symptomatic effects of intra-articular sprifermin in patients with knee osteoarthritis: 5-year results from the FORWARD study. Ann Rheum Dis 80(8):1062–1069. 10.1136/annrheumdis-2020-219181 33962962 10.1136/annrheumdis-2020-219181 PMC8292562

[CR48] Ellsworth JL, Berry J, Bukowski T, Claus J, Feldhaus A, Holderman S, Holdren MS, Lum KD, Moore EE, Raymond F, Ren H, Shea P, Sprecher C, Storey H, Thompson DL, Waggie K, Yao L, Fernandes RJ, Eyre DR, Hughes SD (2002) Fibroblast growth factor-18 is a trophic factor for mature chondrocytes and their progenitors. Osteoarthr Cartil 10(4):308–320. 10.1053/joca.2002.0514 10.1053/joca.2002.0514 11950254

[CR49] Elsaid KA, Zhang L, Shaman Z, Patel C, Schmidt TA, Jay GD (2015) The impact of early intra-articular administration of interleukin-1 receptor antagonist on lubricin metabolism and cartilage degeneration in an anterior cruciate ligament transection model. Osteoarthr Cartil 23(1):114–121. 10.1016/j.joca.2014.09.006 10.1016/j.joca.2014.09.006 PMC4275352 25219670

[CR50] Fasano S, Milone A, Nicoletti GF, Isenberg DA, Ciccia F (2023) Precision medicine in systemic lupus erythematosus. Nat Rev Rheumatol 19(6):331–342. 10.1038/s41584-023-00948-y 37041269 10.1038/s41584-023-00948-y

[CR51] Faust HJ, Zhang H, Han J, Wolf MT, Jeon OH, Sadtler K, Pena AN, Chung L, Maestas DR Jr, Tam AJ, Pardoll DM, Campisi J, Housseau F, Zhou D, Bingham CO 3rd, Elisseeff JH (2020) IL-17 and immunologically induced senescence regulate response to injury in osteoarthritis. J Clin Invest 130(10):5493–5507. 10.1172/JCI134091 32955487 10.1172/JCI134091 PMC7524483

[CR52] Forman HJ, Zhang H, Rinna A (2009) Glutathione: overview of its protective roles, measurement, and biosynthesis. Mol Aspects Med 30(1–2):1–12. 10.1016/j.mam.2008.08.006 18796312 10.1016/j.mam.2008.08.006 PMC2696075

[CR53] Foster NE, Eriksson L, Deveza L, Hall M (2023) Osteoarthritis year in review 2022: epidemiology & therapy. Osteoarthr Cartil 31(7):876–883. 10.1016/j.joca.2023.03.008 10.1016/j.joca.2023.03.008 36963607

[CR54] Furst DE (2004) Anakinra: review of recombinant human interleukin-I receptor antagonist in the treatment of rheumatoid arthritis. Clin Ther 26(12):1960–1975. 10.1016/j.clinthera.2004.12.019 15823761 10.1016/j.clinthera.2004.12.019

[CR55] Gambardella V, Tarazona N, Cejalvo JM, Lombardi P, Huerta M, Roselló S, Fleitas T, Roda D, Cervantes A (2020) Personalized medicine: recent progress in cancer therapy. Cancers 12(4):1009. 10.3390/cancers12041009 32325878 10.3390/cancers12041009 PMC7226371

[CR56] Gandek B, Ware JE Jr (2017) Validity and Responsiveness of the knee injury and osteoarthritis outcome score: a comparative study among total knee replacement patients. Arthritis Care Res (Hoboken) 69(6):817–825. 10.1002/acr.23193 28085998 10.1002/acr.23193 PMC5449223

[CR57] Gigout A, Guehring H, Froemel D, Meurer A, Ladel C, Reker D, Bay-Jensen AC, Karsdal MA, Lindemann S (2017) Sprifermin (rhFGF18) enables proliferation of chondrocytes producing a hyaline cartilage matrix. Osteoarthr Cartil 25(11):1858–1867. 10.1016/j.joca.2017.08.004 10.1016/j.joca.2017.08.004 28823647

[CR58] Gijon-Nogueron G, Balint P, Batalov A, Ostojic P, Sollmann N, Van Middelkoop M, Agricola R, Naili JE, Milovanovic D, Popova S, Kazakova M, Nuernberger S, Aulin C, Karalilova R, Henrotin Y (2025) Terminologies and definitions used to classify patients with osteoarthritis: a scoping review. BMC Rheumatol 9(1):32. 10.1186/s41927-025-00482-2 40087790 10.1186/s41927-025-00482-2 PMC11908012

[CR59] Glasson SS, Askew R, Sheppard B, Carito BA, Blanchet T, Ma HL, Flannery CR, Kanki K, Wang E, Peluso D, Yang Z, Majumdar MK, Morris EA (2004) Characterization of and osteoarthritis susceptibility in ADAMTS-4-knockout mice. Arthritis Rheum 50(8):2547–2558. 10.1002/art.20558 15334469 10.1002/art.20558

[CR60] Glasson SS, Askew R, Sheppard B, Carito B, Blanchet T, Ma HL, Flannery CR, Peluso D, Kanki K, Yang Z, Majumdar MK, Morris EA (2005) Deletion of active ADAMTS5 prevents cartilage degradation in a murine model of osteoarthritis. Nature 434(7033):644–648. 10.1038/nature03369 15800624 10.1038/nature03369

[CR61] Goh GS, Khow YZ, Tay DK, Lo NN, Yeo SJ, Liow MHL (2021) Preoperative mental health influences patient-reported outcome measures and satisfaction after revision total knee arthroplasty. J Arthroplasty 36(8):2878–2886. 10.1016/j.arth.2021.03.026 33812719 10.1016/j.arth.2021.03.026

[CR62] Grillet B, Pereira RVS, Van Damme J, Abu El-Asrar A, Proost P, Opdenakker G (2023) Matrix metalloproteinases in arthritis: towards precision medicine. Nat Rev Rheumatol 19(6):363–377. 10.1038/s41584-023-00966-w 37161083 10.1038/s41584-023-00966-w

[CR63] Grossmann P, Grossmann I (1968) Radiographic findings in successfully treated phosphate diabetes. Radiol Diagn (Berl) 9(2):146–147 5682681

[CR64] Gurumurthy CB, Lloyd KCK (2019) Generating mouse models for biomedical research: technological advances. Dis Model Mech 12(1):dmm029462. 10.1242/dmm.029462 30626588 10.1242/dmm.029462 PMC6361157

[CR65] Hanna FS, Wluka AE, Bell RJ, Davis SR, Cicuttini FM (2004) Osteoarthritis and the postmenopausal woman: epidemiological, magnetic resonance imaging, and radiological findings. Semin Arthritis Rheum 34(3):631–636. 10.1016/j.semarthrit.2004.07.007 15609268 10.1016/j.semarthrit.2004.07.007

[CR66] Hannani MT, Thudium CS, Karsdal MA, Ladel C, Mobasheri A, Uebelhoer M, Larkin J, Bacardit J, Struglics A, Bay-Jensen AC (2024) From biochemical markers to molecular endotypes of osteoarthritis: a review on validated biomarkers. Expert Rev Mol Diagn 24(1–2):23–38. 10.1080/14737159.2024.2315282 38353446 10.1080/14737159.2024.2315282

[CR67] Hart DA (2022) Osteoarthritis as an umbrella term for different subsets of humans undergoing joint degeneration: the need to address the differences to develop effective conservative treatments and prevention strategies. Int J Mol Sci 23(23):15365. 10.3390/ijms232315365 36499704 10.3390/ijms232315365 PMC9736942

[CR68] Hazlewood GS, Barnabe C, Tomlinson G, Marshall D, Devoe DJ, Bombardier C (2016) Methotrexate monotherapy and methotrexate combination therapy with traditional and biologic disease modifying anti-rheumatic drugs for rheumatoid arthritis: a network meta-analysis. Cochrane Database Syst Rev. 10.1002/14651858.CD010227.pub2 27571502 10.1002/14651858.CD010227.pub2 PMC7087436

[CR69] Henrotin Y, Uebelhoer M, Bihlet AR, Donneau AF, Monseur J, Lau E, Müller Bested K, Rovsing H, Rieger F, Nicco C (2024) OP0305 a single intra-articular injection of Jta-004 is safe and efficient for treating symptoms in the most severely affected knee osteoarthritis patients – a post HOC analysis of multicenter, randomized, double-blind, placebo-and active-treatment controlled phase III KOA-2 clinical triaL. Ann Rheum Dis 83(Suppl 1):123. 10.1136/annrheumdis-2024-eular.1910

[CR70] Hochberg MC, Guermazi A, Guehring H, Aydemir A, Wax S, Fleuranceau-Morel P, Reinstrup Bihlet A, Byrjalsen I, Ragnar Andersen J, Eckstein F (2019) Effect of intra-articular sprifermin vs placebo on femorotibial joint cartilage thickness in patients with osteoarthritis: the FORWARD randomized clinical trial. JAMA 322(14):1360–1370. 10.1001/jama.2019.14735 31593273 10.1001/jama.2019.14735 PMC6784851

[CR71] Hsu B, Visich J, Lane NE, Li L, Mittal J, An M, Laberge RM, Dananberg J (2020) Safety, tolerability, pharmacokinetics, and clinical outcomes following treatment of painful knee osteoarthritis with senolytic molecule UBX0101. Osteoarthr Cartil 28:S479–S480. 10.1016/j.joca.2020.02.752

[CR72] Hua B, Qiu J, Ye X, Liu X (2022) Intra-articular injection of a novel Wnt pathway inhibitor, SM04690, upregulates Wnt16 expression and reduces disease progression in temporomandibular joint osteoarthritis. Bone 158:116372. 10.1016/j.bone.2022.116372 35218985 10.1016/j.bone.2022.116372

[CR73] Huang H, Skelly JD, Ayers DC, Song J (2017) Age-dependent changes in the articular cartilage and subchondral bone of C57BL/6 mice after surgical destabilization of medial Meniscus. Sci Rep 7(1):42294. 10.1038/srep42294 28181577 10.1038/srep42294 PMC5299455

[CR74] Hudson MP, Armstrong PW, Ruzyllo W, Brum J, Cusmano L, Krzeski P, Lyon R, Quinones M, Theroux P, Sydlowski D, Kim HE, Garcia MJ, Jaber WA, Weaver WD (2006) Effects of selective matrix metalloproteinase inhibitor (PG-116800) to prevent ventricular remodeling after myocardial infarction: results of the PREMIER (Prevention of Myocardial Infarction Early Remodeling) trial. J Am Coll Cardiol 48(1):15–20. 10.1016/j.jacc.2006.02.055 16814643 10.1016/j.jacc.2006.02.055

[CR75] Hunter DJ & Bierma-Zeinstra S (2019) Osteoarthritis. Lancet 393(10182):1745-1759. 10.1016/S0140-6736(19)30417-9 10.1016/S0140-6736(19)30417-9 31034380

[CR76] Im GI (2022) The concept of early osteoarthritis and its significance in regenerative medicine. Tissue Eng Regen Med 19(3):431–436. 10.1007/s13770-022-00436-6 35244885 10.1007/s13770-022-00436-6 PMC9130436

[CR77] Jallali N, Ridha H, Thrasivoulou C, Underwood C, Butler PE, Cowen T (2005) Vulnerability to ROS-induced cell death in ageing articular cartilage: the role of antioxidant enzyme activity. Osteoarthr Cartil 13(7):614–622. 10.1016/j.joca.2005.02.011 10.1016/j.joca.2005.02.011 15979014

[CR78] Jansen NEJ, Molendijk E, Schiphof D, Van Meurs JBJ, Oei EHG, Van Middelkoop M, Bierma-Zeinstra SMA (2023) Metabolic syndrome and the progression of knee osteoarthritis on MRI. Osteoarthr Cartil 31(5):647–655. 10.1016/j.joca.2023.02.003 10.1016/j.joca.2023.02.003 36801367

[CR79] Jenei-Lanzl Z, Pongratz G (2022) Posttraumatic osteoarthritis as potential modulator of autonomic nervous system function. Osteoarthr Cartil 30(4):498–500. 10.1016/j.joca.2021.12.009 10.1016/j.joca.2021.12.009 35017059

[CR80] Jeon OH, Kim C, Laberge RM, Demaria M, Rathod S, Vasserot AP, Chung JW, Kim DH, Poon Y, David N, Baker DJ, Van Deursen JM, Campisi J, Elisseeff JH (2017) Local clearance of senescent cells attenuates the development of post-traumatic osteoarthritis and creates a pro-regenerative environment. Nat Med 23(6):775–781. 10.1038/nm.4324 28436958 10.1038/nm.4324 PMC5785239

[CR81] Jeon OH, David N, Campisi J, Elisseeff JH (2018) Senescent cells and osteoarthritis: a painful connection. J Clin Invest 128(4):1229–1237. 10.1172/JCI95147 29608139 10.1172/JCI95147 PMC5873863

[CR82] Joos H, Leucht F, Riegger J, Hogrefe C, Fiedler J, durselen l, reichel h, ignatius a, brenner re (2015) differential interactive effects of cartilage traumatization and blood exposure in vitro and in vivo. Am J Sports Med 43(11):2822–2832. 10.1177/0363546515602248 26362437 10.1177/0363546515602248

[CR83] Julovi SM, Dao A, Trinh K, O’Donohue AK, Shu C, Smith S, Shingde M, Schindeler A, Rogers NM, Little CB (2023) Disease-modifying interactions between chronic kidney disease and osteoarthritis: a new comorbid mouse model. RMD Open 9(3):e003109. 10.1136/rmdopen-2023-003109 37562858 10.1136/rmdopen-2023-003109 PMC10423836

[CR84] Kaneko Y, Tanigawa N, Sato Y, Kobayashi T, Nakamura S, Ito E, Soma T, Miyamoto K, Kobayashi S, Harato K, Matsumoto M, Nakamura M, Niki Y, Miyamoto T (2019) Oral administration of N-acetyl cysteine prevents osteoarthritis development and progression in a rat model. Sci Rep 9(1):18741. 10.1038/s41598-019-55297-2 31822750 10.1038/s41598-019-55297-2 PMC6904562

[CR85] Kang D, Lee J, Jung J, Carlson BA, Chang MJ, Chang CB, Kang SB, Lee BC, Gladyshev VN, Hatfield DL, Lee BJ, Kim JH (2022) Selenophosphate synthetase 1 deficiency exacerbates osteoarthritis by dysregulating redox homeostasis. Nat Commun 13(1):779. 10.1038/s41467-022-28385-7 35140209 10.1038/s41467-022-28385-7 PMC8828855

[CR86] Kapoor M, Martel-Pelletier J, Lajeunesse D, Pelletier JP, Fahmi H (2011) Role of proinflammatory cytokines in the pathophysiology of osteoarthritis. Nat Rev Rheumatol 7(1):33–42. 10.1038/nrrheum.2010.196 21119608 10.1038/nrrheum.2010.196

[CR87] Karsdal MA, Christiansen C, Ladel C, Henriksen K, Kraus VB, Bay-Jensen AC (2014) Osteoarthritis–a case for personalized health care? Osteoarthr Cartil 22(1):7–16. 10.1016/j.joca.2013.10.018 10.1016/j.joca.2013.10.018 24216058

[CR88] Karsdal MA, Tambiah J, Felson D, Ladel C, Nikolov NP, Hodgins D, Bihlet AR, Neogi T, Baatenburg De Jong C, Bay-Jensen AC, Baron R, Laslop A, Mobasheri A, Kraus VB (2023) Reflections from the OARSI 2022 clinical trials symposium: the pain of OA-Deconstruction of pain and patient-reported outcome measures for the benefit of patients and clinical trial design. Osteoarthr Cartil 31(10):1293–1302. 10.1016/j.joca.2023.06.006 10.1016/j.joca.2023.06.006 PMC11184959 37380011

[CR89] Kim H, Seo J, Lee Y, Park K, Perry TA, Arden NK, Mobasheri A, Choi H (2022) The current state of the osteoarthritis drug development pipeline: a comprehensive narrative review of the present challenges and future opportunities. Ther Adv Musculoskelet Dis. 10.1177/1759720X221085952 36504595 10.1177/1759720X221085952 PMC9732806

[CR90] Kirkland JL, Tchkonia T (2020) Senolytic drugs: from discovery to translation. J Intern Med 288(5):518–536. 10.1111/joim.13141 32686219 10.1111/joim.13141 PMC7405395

[CR91] Kittelson AJ, Stevens-Lapsley JE, Schmiege SJ (2016) Determination of pain phenotypes in knee osteoarthritis: a latent class analysis using data from the osteoarthritis initiative. Arthritis Care Res (Hoboken) 68(5):612–620. 10.1002/acr.22734 26414884 10.1002/acr.22734 PMC5388442

[CR92] Knoop J, Van Der Leeden M, Thorstensson CA, Roorda LD, Lems WF, Knol DL, Steultjens MP, Dekker J (2011) Identification of phenotypes with different clinical outcomes in knee osteoarthritis: data from the Osteoarthritis Initiative. Arthritis Care Res (Hoboken) 63(11):1535–1542. 10.1002/acr.20571 21954070 10.1002/acr.20571

[CR93] Kotimaa J, Klar-Mohammad N, Gueler F, Schilders G, Jansen A, Rutjes H, Daha MR, van Kooten C (2016) Sex matters: systemic complement activity of female C57BL/6J and BALB/cJ mice is limited by serum terminal pathway components. Mol Immunol 76:13–21. 10.1016/j.molimm.2016.06.004 27337595 10.1016/j.molimm.2016.06.004

[CR94] Kraus NR, Lowenstein NA, Garvey KD, Matzkin EG (2021) Smoking negatively effects patient-reported outcomes following arthroscopic partial meniscectomy. Arthrosc Sports Med Rehabil 3(2):e323–e328. 10.1016/j.asmr.2020.09.021 34027438 10.1016/j.asmr.2020.09.021 PMC8129051

[CR95] Krueger DR, Leopold VJ, Schroeder JH, Perka C, Hardt S (2020) Correlation of the subjective hip value with validated patient-reported outcome measurements for the hip. J Clin Med 9(7):2179. 10.3390/jcm9072179 32664255 10.3390/jcm9072179 PMC7409009

[CR96] Krzeski P, Buckland-Wright C, Balint G, Cline GA, Stoner K, Lyon R, Beary J, Aronstein WS, Spector TD (2007) Development of musculoskeletal toxicity without clear benefit after administration of PG-116800, a matrix metalloproteinase inhibitor, to patients with knee osteoarthritis: a randomized, 12-month, double-blind, placebo-controlled study. Arthritis Res Ther 9(5):R109. 10.1186/ar2315 17958901 10.1186/ar2315 PMC2212568

[CR97] Kuswanto W, Baker MC (2024) Repurposing drugs for the treatment of osteoarthritis. Osteoarthr Cartil 32(8):886–895. 10.1016/j.joca.2024.05.008 10.1016/j.joca.2024.05.008 38821468

[CR98] Lane N, Hsu B, Visich J, Xie B, Khan A, Dananberg J (2021) A phase 2, randomized, double-blind, placebo-controlled study of senolytic molecule UBX0101 in the treatment of painful knee osteoarthritis. Osteoarthr Cartil 29:S52–S53

[CR99] Latourte A, Kloppenburg M, Richette P (2020) Emerging pharmaceutical therapies for osteoarthritis. Nat Rev Rheumatol 16(12):673–688. 10.1038/s41584-020-00518-6 33122845 10.1038/s41584-020-00518-6

[CR100] Leopold VJ, Homm PM, Kruger D, Hipfl C, Perka C, Hardt S (2024) The subjective hip value is a valid, reliable, and responsive instrument for assessing hip function in primary total hip arthroplasty. J Arthroplast 39(7):1789–1795. 10.1016/j.arth.2024.01.061 10.1016/j.arth.2024.01.061 38336302

[CR101] Lepescheux L, Clement-Lacroix P, Merciris D, Meurisse S, Borgonovi M, Cottereaux C, Mollat P, Brebion F, Gosmini R, De Ceuninck F, Botez I, Van Der Aar E, Christophe T, Vandervoort N, Blanqué R, Comas D, Deprez P, Amantini D (2018) OP0258 Efficacy of the highly selective adamts-5 inhibitor glpg1972 in the rat meniscectomy model. Ann Rheum Dis 77(Suppl 2):178. 10.1136/annrheumdis-2018-eular.2610

[CR102] Lewis MJ, Barnes MR, Blighe K, Goldmann K, Rana S, Hackney JA, Ramamoorthi N, John CR, Watson DS, Kummerfeld SK, Hands R (2019) Molecular portraits of early rheumatoid arthritis identify clinical and treatment response phenotypes. Cell Rep 28(9):2455–2470. 10.1016/j.celrep.2019.07.091 31461658 10.1016/j.celrep.2019.07.091 PMC6718830

[CR103] Li NG, Shi ZH, Tang YP, Wang ZJ, Song SL, Qian LH, Qian DW, Duan JA (2011) New hope for the treatment of osteoarthritis through selective inhibition of MMP-13. Curr Med Chem 18(7):977–1001. 10.2174/092986711794940905 21254976 10.2174/092986711794940905

[CR104] Li X, Han Y, Li G, Zhang Y, Wang J, Feng C (2023a) Role of Wnt signaling pathway in joint development and cartilage degeneration. Front Cell Dev Biol 11:1181619. 10.3389/fcell.2023.1181619 37363728 10.3389/fcell.2023.1181619 PMC10285172

[CR105] Li Y, Tang JS, Zhou ZS, Wang CY, Peng YC, Zuo JL (2023b) Quantitative study of 30T MRI on the thickness of knee joint cartilage in healthy young people. Zhongguo Gu Shang 36(11):1065–1069. 10.12200/j.issn.1003-0034.2023.11.011 38012876 10.12200/j.issn.1003-0034.2023.11.011

[CR106] Li ZA, Sant S, Cho SK, Goodman SB, Bunnell BA, Tuan RS, Gold MS, Lin H (2023c) Synovial joint-on-a-chip for modeling arthritis: progress, pitfalls, and potential. Trends Biotechnol 41(4):511–527. 10.1016/j.tibtech.2022.07.011 35995600 10.1016/j.tibtech.2022.07.011 PMC9938846

[CR107] Liao Y, Ren Y, Luo X, Mirando AJ, Long JT, Leinroth A, Ji RR, Hilton MJ (2022) Interleukin-6 signaling mediates cartilage degradation and pain in posttraumatic osteoarthritis in a sex-specific manner. Sci Signal 15(744):eabn7082. 10.1126/scisignal.abn7082 35881692 10.1126/scisignal.abn7082 PMC9382892

[CR108] Little CB, Barai A, Burkhardt D, Smith SM, Fosang AJ, Werb Z, Shah M, Thompson EW (2009) Matrix metalloproteinase 13-deficient mice are resistant to osteoarthritic cartilage erosion but not chondrocyte hypertrophy or osteophyte development. Arthritis Rheum 60(12):3723–3733. 10.1002/art.25002 19950295 10.1002/art.25002 PMC2832925

[CR109] Little CB, Zaki S, Blaker CL, Clarke EC (2022) Animal models of osteoarthritis: the benefits and costs of reducing variability. Bone Joint Res 11(8):514–517. 10.1302/2046-3758.118.BJR-2022-0217.R1 35909339 10.1302/2046-3758.118.BJR-2022-0217.R1 PMC9396918

[CR110] Loeser RF, Goldring SR, Scanzello CR, Goldring MB (2012) Osteoarthritis: a disease of the joint as an organ. Arthritis Rheum 64(6):1697–1707. 10.1002/art.34453 22392533 10.1002/art.34453 PMC3366018

[CR111] Lohmander LS, Hellot S, Dreher D, Krantz EF, Kruger DS, Guermazi A, Eckstein F (2014) Intraarticular sprifermin (recombinant human fibroblast growth factor 18) in knee osteoarthritis: a randomized, double-blind, placebo-controlled trial. Arthritis Rheumatol 66(7):1820–1831. 10.1002/art.38614 24740822 10.1002/art.38614

[CR112] Longo UG, Papalia R, De Salvatore S, Picozzi R, Sarubbi A, Denaro V (2023) Induced models of osteoarthritis in animal models: a systematic review. Biol 12(2):283. 10.3390/biology12020283 10.3390/biology12020283 PMC9953428 36829562

[CR113] Lopez J, Al-Nakkash L, Broderick TL, Castro M, Tobin B, Plochocki JH (2023) Genistein suppresses IL-6 and MMP-13 to attenuate osteoarthritis in obese diabetic mice. Metabolites 13(9):1014. 10.3390/metabo13091014 37755294 10.3390/metabo13091014 PMC10534591

[CR114] Lorenz J, Grässel S (2014) Experimental osteoarthritis models in mice. Mouse Genet: Methods Protoc. 10.1007/978-1-4939-1215-5_23 10.1007/978-1-4939-1215-5_23 25064117

[CR115] Lories RJ, Monteagudo S (2020) Review article: is wnt signaling an attractive target for the treatment of osteoarthritis? Rheumatol Ther 7(2):259–270. 10.1007/s40744-020-00205-8 32277404 10.1007/s40744-020-00205-8 PMC7211213

[CR116] Lundgren-Nilsson Å, Dencker A, Palstam A, Person G, Horton MC, Escorpizo R, Küçükdeveci AA, Kutlay S, Elhan AH, Stucki G, Tennant A, Conaghan PG (2018) Patient-reported outcome measures in osteoarthritis: a systematic search and review of their use and psychometric properties. RMD Open 4(2):e000715. 10.1136/rmdopen-2018-000715 30622735 10.1136/rmdopen-2018-000715 PMC6307597

[CR117] Luo Y, Samuels J, Krasnokutsky S, Byrjalsen I, Kraus VB, He Y, Karsdal MA, Abramson SB, Attur M, Bay-Jensen AC (2021) A low cartilage formation and repair endotype predicts radiographic progression of symptomatic knee osteoarthritis. J Orthop Traumatol 22(1):10. 10.1186/s10195-021-00572-0 33687578 10.1186/s10195-021-00572-0 PMC7943687

[CR118] Luyten FP, Bierma-Zeinstra S, Dell’accio F, Kra us VB, Nakata K, Sekiya I, Arden NK, Lohmander LS (2018) Toward classification criteria for early osteoarthritis of the knee. Semin Arthritis Rheum 47(4):457–463. 10.1016/j.semarthrit.2017.08.006 28917712 10.1016/j.semarthrit.2017.08.006

[CR119] Ma CH, Lv Q, Yu YX, Zhang Y, Kong D, Niu KR, Yi CQ (2015) Protective effects of tumor necrosis factor-alpha blockade by adalimumab on articular cartilage and subchondral bone in a rat model of osteoarthritis. Braz J Med Biol Res 48(10):863–870. 10.1590/1414-431X20154407 26445328 10.1590/1414-431X20154407 PMC4617111

[CR120] Madry H, Kon E, Condello V, Peretti GM, Steinwachs M, Seil R, Berruto M, Engebretsen L, Filardo G, Angele P (2016) Early osteoarthritis of the knee. Knee Surg Sports Traumatol Arthrosc 24(6):1753–1762. 10.1007/s00167-016-4068-3 27000393 10.1007/s00167-016-4068-3

[CR121] Mahmoudian A, Lohmander LS, Jafari H, Luyten FP (2021a) Towards classification criteria for early-stage knee osteoarthritis: a population-based study to enrich for progressors. Semin Arthritis Rheum 51(1):285–291. 10.1016/j.semarthrit.2020.11.002 33433364 10.1016/j.semarthrit.2020.11.002

[CR122] Mahmoudian A, Lohmander LS, Mobasheri A, Englund M, Luyten FP (2021b) Early-stage symptomatic osteoarthritis of the knee - time for action. Nat Rev Rheumatol 17(10):621–632. 10.1038/s41584-021-00673-4 34465902 10.1038/s41584-021-00673-4

[CR123] Majumdar MK, Askew R, Schelling S, Stedman N, Blanchet T, Hopkins B, Morris EA, Glasson SS (2007) Double-knockout of ADAMTS-4 and ADAMTS-5 in mice results in physiologically normal animals and prevents the progression of osteoarthritis. Arthritis Rheum 56(11):3670–3674. 10.1002/art.23027 17968948 10.1002/art.23027

[CR124] Manivong S, Cullier A, Audigie F, Banquy X, Moldovan F, Demoor M, Roullin VG (2023) New trends for osteoarthritis: biomaterials, models and modeling. Drug Discov Today 28(3):103488. 10.1016/j.drudis.2023.103488 36623796 10.1016/j.drudis.2023.103488

[CR125] Mccoy AM (2015) Animal models of osteoarthritis: comparisons and key considerations. Vet Pathol 52(5):803–818. 10.1177/0300985815588611 26063173 10.1177/0300985815588611

[CR126] Mcculloch K, Litherland GJ, Rai TS (2017) Cellular senescence in osteoarthritis pathology. Aging Cell 16(2):210–218. 10.1111/acel.12562 28124466 10.1111/acel.12562 PMC5334539

[CR127] Mcnulty MA, Loeser RF, Davey C, Callahan MF, Ferguson CM, Carlson CS (2012) Histopathology of naturally occurring and surgically induced osteoarthritis in mice. Osteoarthr Cartil 20(8):949–956. 10.1016/j.joca.2012.05.001 10.1016/j.joca.2012.05.001 PMC3402508 22595226

[CR128] Migliore A, Scire CA, Carmona L, Herrero-Beaumont G, Bizzi E, Branco J, Carrara G, Chevalier X, Collaku L, Aslanidis S, Denisov L, Di Matteo L, Bianchi G, Diracoglu D, Frediani B, Maheu E, Martusevich N, Bagnato GF, Scarpellini M, Minisola G, Akkoc N, Ramonda R, Barskova T, Babic-Naglic D, Muelas JVM, Ionescu R, Rashkov R, Damjanov N, Cerinic MM (2017) The challenge of the definition of early symptomatic knee osteoarthritis: a proposal of criteria and red flags from an international initiative promoted by the Italian Society for Rheumatology. Rheumatol Int 37(8):1227–1236. 10.1007/s00296-017-3700-y 28451793 10.1007/s00296-017-3700-y

[CR129] Migliorini F, Driessen A, Quack V, Sippel N, Cooper B, Mansy YE, Tingart M, Eschweiler J (2021) Comparison between intra-articular infiltrations of placebo, steroids, hyaluronic and PRP for knee osteoarthritis: a Bayesian network meta-analysis. Arch Orthop Trauma Surg 141(9):1473–1490. 10.1007/s00402-020-03551-y 32725315 10.1007/s00402-020-03551-y

[CR130] Miller RE, Miller RJ, Malfait AM (2014) Osteoarthritis joint pain: the cytokine connection. Cytokine 70(2):185–193. 10.1016/j.cyto.2014.06.019 25066335 10.1016/j.cyto.2014.06.019 PMC4254338

[CR131] Mobasheri A, Loeser R (2024) Clinical phenotypes, molecular endotypes and theratypes in OA therapeutic development. Nat Rev Rheumatol 20(9):525–526. 10.1038/s41584-024-01126-4 38760581 10.1038/s41584-024-01126-4

[CR132] Mobasheri A, Rayman MP, Gualillo O, Sellam J, Van Der Kraan P, Fearon U (2017) The role of metabolism in the pathogenesis of osteoarthritis. Nat Rev Rheumatol 13(5):302–311. 10.1038/nrrheum.2017.50 28381830 10.1038/nrrheum.2017.50

[CR133] Mobasheri A, Van Spil WE, Budd E, Uzieliene I, Bernotiene E, Bay-Jensen AC, Larkin J, Levesque MC, Gualillo O, Henrotin Y (2019) Molecular taxonomy of osteoarthritis for patient stratification, disease management and drug development: biochemical markers associated with emerging clinical phenotypes and molecular endotypes. Curr Opin Rheumatol 31(1):80–89. 10.1097/BOR.0000000000000567 30461544 10.1097/BOR.0000000000000567

[CR134] Mobasheri A, Kapoor M, Ali SA, Lang A, Madry H (2021) The future of deep phenotyping in osteoarthritis: how can high throughput omics technologies advance our understanding of the cellular and molecular taxonomy of the disease? Osteoarthr Cartil Open 3(4):100144. 10.1016/j.ocarto.2021.100144 36474763 10.1016/j.ocarto.2021.100144 PMC9718223

[CR135] Mohan G, Perilli E, Kuliwaba JS, Humphries JM, Parkinson IH, Fazzalari NL (2011) Application of in vivo micro-computed tomography in the temporal characterisation of subchondral bone architecture in a rat model of low-dose monosodium iodoacetate-induced osteoarthritis. Arthritis Res Ther 13(6):R210. 10.1186/ar3543 22185204 10.1186/ar3543 PMC3334663

[CR136] Molnar V, Matišić V, Kodvanj I, Bjelica R, Jeleč Ž, Hudetz D, Rod E, Čukelj F, Vrdoljak T, Vidović D, Starešinić M (2021) Cytokines and chemokines involved in osteoarthritis pathogenesis. Int J Mol Sci 22(17):9208. 10.3390/ijms22179208 34502117 10.3390/ijms22179208 PMC8431625

[CR137] Momtazmanesh S, Nowroozi A, Rezaei N (2022) Artificial intelligence in rheumatoid arthritis: current status and future perspectives: a state-of-the-art review. Rheumatol Ther 9(5):1249–1304. 10.1007/s40744-022-00475-4 35849321 10.1007/s40744-022-00475-4 PMC9510088

[CR138] Mooney RA, Sampson ER, Lerea J, Rosier RN, Zuscik MJ (2011) High-fat diet accelerates progression of osteoarthritis after meniscal/ligamentous injury. Arthritis Res Ther 13(6):R198. 10.1186/ar3529 22152451 10.1186/ar3529 PMC3334649

[CR139] Moore EE, Bendele AM, Thompson DL, Littau A, Waggie KS, Reardon B, Ellsworth JL (2005) Fibroblast growth factor-18 stimulates chondrogenesis and cartilage repair in a rat model of injury-induced osteoarthritis. Osteoarthr Cartil 13(7):623–631. 10.1016/j.joca.2005.03.003 10.1016/j.joca.2005.03.003 15896984

[CR140] Moreau S, Flores-Berdines R, Jalkh TE, Simon A, Taret G, Fomina A, Dargenet-Becker C, Estevez-Torres A, Bernard S, Salmon H (2024) Reversible and reusable compartmentalized microfluidic chip for coculture of dorsal root ganglion neurons. Biorxiv. 10.1101/2024.12.10.62713939651154

[CR141] Mori Y, Saito T, Chang SH, Kobayashi H, Ladel CH, Guehring H, Chung UI, Kawaguchi H (2014) Identification of fibroblast growth factor-18 as a molecule to protect adult articular cartilage by gene expression profiling. J Biol Chem 289(14):10192–10200. 10.1074/jbc.M113.524090 24577103 10.1074/jbc.M113.524090 PMC3974988

[CR142] Mylonas A, O’Loghlen A (2022) Cellular senescence and ageing: mechanisms and interventions. Front Aging 3:866718. 10.3389/fragi.2022.866718 35821824 10.3389/fragi.2022.866718 PMC9261318

[CR143] Nakagawa S, Arai Y, Mazda O, Kishida T, Takahashi KA, Sakao K, Saito M, Honjo K, Imanishi J, Kubo T (2010) N-acetylcysteine prevents nitric oxide-induced chondrocyte apoptosis and cartilage degeneration in an experimental model of osteoarthritis. J Orthop Res 28(2):156–163. 10.1002/jor.20976 19725096 10.1002/jor.20976

[CR144] Nalesso G, Thomas BL, Sherwood JC, Yu J, Addimanda O, Eldridge SE, Thorup AS, Dale L, Schett G, Zwerina J, Eltawil N, Pitzalis C, Dell’accio F (2017) WNT16 antagonises excessive canonical WNT activation and protects cartilage in osteoarthritis. Ann Rheum Dis 76(1):218–226. 10.1136/annrheumdis-2015-208577 27147711 10.1136/annrheumdis-2015-208577 PMC5264226

[CR145] Neuhold LA, Killar L, Zhao W, Sung ML, Warner L, Kulik J, Turner J, Wu W, Billinghurst C, Meijers T, Poole AR, Babij P, Degennaro LJ (2001) Postnatal expression in hyaline cartilage of constitutively active human collagenase-3 (MMP-13) induces osteoarthritis in mice. J Clin Invest 107(1):35–44. 10.1172/JCI10564 11134178 10.1172/JCI10564 PMC198546

[CR146] O’connor SK, Katz DB, Oswald SJ, Groneck L, Guilak F (2021) Formation of osteochondral organoids from murine induced pluripotent stem cells. Tissue Eng Part A 27(15–16):1099–1109. 10.1089/ten.TEA.2020.0273 33191853 10.1089/ten.tea.2020.0273 PMC8392116

[CR147] Oo WM, Hunter DJ (2022) Repurposed and investigational disease-modifying drugs in osteoarthritis (DMOADs). Ther Adv Musculoskelet Dis. 10.1177/1759720X221090297 35619876 10.1177/1759720X221090297 PMC9128067

[CR148] Oo WM, Little C, Duong V, Hunter DJ (2021) The development of disease-modifying therapies for osteoarthritis (DMOADs): the evidence to date. Drug des Devel Ther 15:2921–2945. 10.2147/DDDT.S295224 34262259 10.2147/DDDT.S295224 PMC8273751

[CR149] Orr MN, Klika AK, Emara AK, Piuzzi NS, Higuera-Rueda CA, Barsoum WK, Molloy RM, Murray TG, Krebs VE, Patel PD, Stearns KL (2022) Combinations of preoperative patient-reported outcome measure phenotype (Pain, Function, and Mental Health) predict outcome after total knee arthroplasty. The J Arthroplast 37(6):S110–S120. 10.1016/j.arth.2022.02.090 10.1016/j.arth.2022.02.090 35240283

[CR150] Ozcamdalli M, Misir A, Kizkapan TB, Uzun E, Duygulu F, Yazici C, Kafadar IH (2017) Comparison of intra-articular injection of hyaluronic acid and N-Acetyl cysteine in the treatment of knee osteoarthritis: a pilot study. Cartilage 8(4):384–390. 10.1177/1947603516675915 28934876 10.1177/1947603516675915 PMC5613896

[CR151] Perruccio AV, Chandran V, Power JD, Kapoor M, Mahomed NN, Gandhi R (2017) Systemic inflammation and painful joint burden in osteoarthritis: a matter of sex? Osteoarthr Cartil 25(1):53–59. 10.1016/j.joca.2016.08.001 10.1016/j.joca.2016.08.001 27546883

[CR152] Perruccio AV, Badley EM, Power JD, Canizares M, Kapoor M, Rockel J, Chandran V, Gandhi R, Mahomed NM, Davey JR, Syed K, Veillette C, Rampersaud YR (2019) Sex differences in the relationship between individual systemic markers of inflammation and pain in knee osteoarthritis. Osteoarthr Cartil Open 1(1–2):100004. 10.1016/j.ocarto.2019.100004 36474721 10.1016/j.ocarto.2019.100004 PMC9718173

[CR153] Perruccio AV, Fitzpatrick J, Power JD, Gandhi R, Rampersaud YR, Mahomed NN, Davey JR, Syed K, Veillette C, Badley EM (2020) Sex-modified effects of depression, low back pain, and comorbidities on pain after total knee arthroplasty for osteoarthritis. Arthritis Care Res (Hoboken) 72(8):1074–1080. 10.1002/acr.24002 31199582 10.1002/acr.24002

[CR154] Plachel F, Jung T, Bartek B, Ruttershoff K, Perka C, Gwinner C (2022) The subjective knee value is a valid single-item survey to assess knee function in common knee disorders. Arch Orthop Trauma Surg 142(8):1723–1730. 10.1007/s00402-021-03794-3 33523264 10.1007/s00402-021-03794-3 PMC9296395

[CR155] Poulsen RC, Jain L, Dalbeth N (2023) Re-thinking osteoarthritis pathogenesis: what can we learn (and what do we need to unlearn) from mouse models about the mechanisms involved in disease development. Arthritis Res Ther 25(1):59. 10.1186/s13075-023-03042-6 37046337 10.1186/s13075-023-03042-6 PMC10100340

[CR156] Power J, Hernandez P, Guehring H, Getgood A, Henson F (2014) Intra-articular injection of rhFGF-18 improves the healing in microfracture treated chondral defects in an ovine model. J Orthop Res 32(5):669–676. 10.1002/jor.22580 24436147 10.1002/jor.22580

[CR157] Rai MF, Duan X, Quirk JD, Holguin N, Schmidt EJ, Chinzei N, Silva MJ, Sandell LJ (2017) Post-traumatic osteoarthritis in mice following mechanical injury to the synovial joint. Sci Rep 7:545223. 10.1038/srep45223 10.1038/srep45223 PMC5366938 28345597

[CR158] Rai MF, Collins KH, Lang A, Maerz T, Geurts J, Ruiz-Romero C, June RK, Ramos Y, Rice SJ, Ali SA, Pastrello C, Jurisica I, Thomas Appleton C, Rockel JS, Kapoor M (2023) Three decades of advancements in osteoarthritis research: insights from transcriptomic, proteomic, and metabolomic studies. Osteoarthr Cartil. 10.1016/j.joca.2023.11.019 10.1016/j.joca.2023.11.019 PMC12239761 38049029

[CR159] Riegger J, Brenner RE (2020) Pathomechanisms of posttraumatic osteoarthritis: chondrocyte behavior and fate in a precarious environment. Int J Mol Sci 21(5):1560. 10.3390/ijms21051560 32106481 10.3390/ijms21051560 PMC7084733

[CR160] Riegger J, Leucht F, Palm HG, Ignatius A, Brenner RE (2019) Initial harm reduction by n-acetylcysteine alleviates cartilage degeneration after blunt single-impact cartilage trauma in vivo. Int J Mol Sci 20(12):2916. 10.3390/ijms20122916 31207966 10.3390/ijms20122916 PMC6628290

[CR161] Riegger J, Huber-Lang M, Brenner RE (2020) Crucial role of the terminal complement complex in chondrocyte death and hypertrophy after cartilage trauma. Osteoarthr Cartil 28(5):685–697. 10.1016/j.joca.2020.01.004 10.1016/j.joca.2020.01.004 31981738

[CR162] Riegger J, Joos H, Mohler V, Leucht F, Rading K, Kubisch C, Ignatius A, Huber-Lang M, Brenner RE (2023a) Functional loss of terminal complement complex protects rabbits from injury-induced osteoarthritis on structural and cellular level. Biomolecules 13:2. 10.3390/biom13020216 10.3390/biom13020216 PMC9953363 36830586

[CR163] Riegger J, Schoppa A, Ruths L, Haffner-Luntzer M, Ignatius A (2023b) Oxidative stress as a key modulator of cell fate decision in osteoarthritis and osteoporosis: a narrative review. Cell Mol Biol Lett 28(1):76. 10.1186/s11658-023-00489-y 37777764 10.1186/s11658-023-00489-y PMC10541721

[CR164] Ritter J, Menger M, Herath SC, Histing T, Kolbenschlag J, Daigeler A, Heinzel JC, Prahm C (2023) Translational evaluation of gait behavior in rodent models of arthritic disorders with the CatWalk device–a narrative review. Front Med 10:1255215. 10.3389/fmed.2023.1255215 10.3389/fmed.2023.1255215 PMC10587608 37869169

[CR165] Ro JY, Zhang Y, Tricou C, Yang D, Da Silva JT, Zhang R (2020) Age and sex differences in acute and osteoarthritis-like pain responses in rats. J Gerontol A Biol Sci Med Sci 75(8):1465–1472. 10.1093/gerona/glz186 31412104 10.1093/gerona/glz186 PMC7357590

[CR166] Rodriguez-Merchan EC (2023) The current role of disease-modifying osteoarthritis drugs. Arch Bone Jt Surg 11(1):11–22. 10.22038/ABJS.2021.56530.2807 36793668 10.22038/ABJS.2021.56530.2807 PMC9903308

[CR167] Roemer FW, Aydemir A, Lohmander S, Crema MD, Marra MD, Muurahainen N, Felson DT, Eckstein F, Guermazi A (2016) Structural effects of sprifermin in knee osteoarthritis: a post-hoc analysis on cartilage and non-cartilaginous tissue alterations in a randomized controlled trial. BMC Musculoskelet Disord 17:267. 10.1186/s12891-016-1128-2 27393009 10.1186/s12891-016-1128-2 PMC4938999

[CR168] Rothbauer M, Reihs EI, Fischer A, Windhager R, Jenner F, Toegel S (2022) A progress report and roadmap for microphysiological systems and organ-on-a-chip technologies to be more predictive models in human (knee) osteoarthritis. Front Bioeng Biotechnol 10:886360. 10.3389/fbioe.2022.886360 35782494 10.3389/fbioe.2022.886360 PMC9240813

[CR169] Ruths L, Huber-Lang M, Schulze-Tanzil G, Riegger J (2024) Anaphylatoxins and their corresponding receptors as potential drivers in cartilage calcification during osteoarthritis progression. Osteoarthr Cartil. 10.1016/j.joca.2024.01.004 10.1016/j.joca.2024.01.004 38242312

[CR170] Samuni Y, Goldstein S (1830) Dean OM & Berk M (2013) the chemistry and biological activities of N-acetylcysteine. Biochim Biophys Acta 8:4117–4129. 10.1016/j.bbagen.2013.04.016 10.1016/j.bbagen.2013.04.016 23618697

[CR171] Saxer F, Hollinger A, Bjurstrom MF, Conaghan PG, Neogi T, Schieker M, Berenbaum F (2024) Pain-phenotyping in osteoarthritis: Current concepts, evidence, and considerations towards a comprehensive framework for assessment and treatment. Osteoarthr Cartil Open 6(1):100433. 10.1016/j.ocarto.2023.100433 38225987 10.1016/j.ocarto.2023.100433 PMC10788802

[CR172] Schnitzer T, Pueyo M, Deckx H, Van Der Aar E, Bernard K, Hatch S, Van Der Stoep M, Grankov S, Phung D, Imbert O, Chimits D, Muller K, Hochberg MC, Bliddal H, Wirth W, Eckstein F, Conaghan PG (2023) Evaluation of S201086/GLPG1972, an ADAMTS-5 inhibitor, for the treatment of knee osteoarthritis in ROCCELLA: a phase 2 randomized clinical trial. Osteoarthr Cartil 31(7):985–994. 10.1016/j.joca.2023.04.001 10.1016/j.joca.2023.04.001 37059327

[CR173] Schoettler N, Strek ME (2020) Recent advances in severe asthma: from phenotypes to personalized medicine. Chest 157(3):516–528. 10.1016/j.chest.2019.10.009 31678077 10.1016/j.chest.2019.10.009 PMC7609962

[CR174] Segal NA, Nilges JM, Oo WM (2024) Sex differences in osteoarthritis prevalence, pain perception, physical function and therapeutics. Osteoarthr Cartil. 10.1016/j.joca.2024.04.002 10.1016/j.joca.2024.04.002 38588890

[CR175] Serra CI, Soler C (2019) Animal models of osteoarthritis in small mammals. Vet Clin North Am Exot Anim Pract 22(2):211–221. 10.1016/j.cvex.2019.01.004 30961898 10.1016/j.cvex.2019.01.004

[CR176] Settle S, Vickery L, Nemirovskiy O, Vidmar T, Bendele A, Messing D, Ruminski P, Schnute M, Sunyer T (2010) Cartilage degradation biomarkers predict efficacy of a novel, highly selective matrix metalloproteinase 13 inhibitor in a dog model of osteoarthritis: confirmation by multivariate analysis that modulation of type II collagen and aggrecan degradation peptides parallels pathologic changes. Arthritis Rheum 62(10):3006–3015. 10.1002/art.27596 20533541 10.1002/art.27596

[CR177] Sheets DW Jr, Okamoto T, Dijkgraaf LC, Milam SB, Schmitz JP, Zardeneta G (2006) Free radical damage in facsimile synovium: correlation with adhesion formation in osteoarthritic TMJs. J Prosthodont 15(1):9–19. 10.1111/j.1532-849X.2006.00063.x 16433646 10.1111/j.1532-849X.2006.00063.x

[CR178] Siebuhr AS, Werkmann D, Bay-Jensen AC, Thudium CS, Karsdal MA, Serruys B, Ladel C, Michaelis M, Lindemann S (2020) The anti-ADAMTS-5 nanobody® M6495 protects cartilage degradation ex vivo. Int J Mol Sci 21(17):5992. 10.3390/ijms21175992 32825512 10.3390/ijms21175992 PMC7503673

[CR179] Sohn R, Jenei-Lanzl Z (2023) Role of the sympathetic nervous system in mild chronic inflammatory diseases: focus on osteoarthritis. NeuroImmunoModulation 30(1):143–166. 10.1159/000531798 37429263 10.1159/000531798 PMC10428144

[CR180] Song RH, Tortorella MD, Malfait AM, Alston JT, Yang Z, Arner EC, Griggs DW (2007) Aggrecan degradation in human articular cartilage explants is mediated by both ADAMTS-4 and ADAMTS-5. Arthritis Rheum 56(2):575–585. 10.1002/art.22334 17265492 10.1002/art.22334

[CR181] Soul J, Dunn SL, Anand S, Serracino-Inglott F, Schwartz JM, Boot-Handford RP, Hardingham TE (2018) Stratification of knee osteoarthritis: two major patient subgroups identified by genome-wide expression analysis of articular cartilage. Ann Rheum Dis 77(3):423. 10.1136/annrheumdis-2017-212603 29273645 10.1136/annrheumdis-2017-212603 PMC5867416

[CR182] Speed TJ, Richards JM, Finan PH, Smith MT (2017) Sex moderates the effects of positive and negative affect on clinical pain in patients with knee osteoarthritis. Scand J Pain. 10.1016/j.sjpain.2017.03.005 28850415 10.1016/j.sjpain.2017.03.005 PMC5576503

[CR183] Stampella A, Monteagudo S, Lories R (2017) Wnt signaling as target for the treatment of osteoarthritis. Best Pract Res Clin Rheumatol 31(5):721–729. 10.1016/j.berh.2018.03.004 30509416 10.1016/j.berh.2018.03.004

[CR184] Stanton H, Rogerson FM, East CJ, Golub SB, Lawlor KE, Meeker CT, Little CB, Last K, Farmer PJ, Campbell IK, Fourie AM, Fosang AJ (2005) ADAMTS5 is the major aggrecanase in mouse cartilage in vivo and in vitro. Nature 434(7033):648–652. 10.1038/nature03417 15800625 10.1038/nature03417

[CR185] Sun D, Gao W, Hu H, Zhou S (2022) Why 90% of clinical drug development fails and how to improve it? Acta Pharm Sin B 12(7):3049–3062. 10.1016/j.apsb.2022.02.002 35865092 10.1016/j.apsb.2022.02.002 PMC9293739

[CR186] Swain S, Coupland C, Strauss V, Mallen C, Kuo CF, Sarmanova A, Bierma-Zeinstra SMA, Englund M, Prieto-Alhambra D, Doherty M, Zhang W (2022) Clustering of comorbidities and associated outcomes in people with osteoarthritis - A UK Clinical Practice Research Datalink study. Osteoarthr Cartil 30(5):702–713. 10.1016/j.joca.2021.12.013 10.1016/j.joca.2021.12.013 35122943

[CR187] Swearingen C, Yazici Y, Tambiah JRS, Conaghan PG (2024) POS1177 Treatment with lorecivivint leads to improved long-term patient acceptable symptom state (PASS) compared to placebo: Data from Pahse 3 extentsion trial. Ann Rheum Dis 83(Suppl 1):670–671. 10.1136/annrheumdis-2024-eular.5875

[CR188] Swearingen CJ, Tambiah JRS, Simsek I, Ghandehari H, Kennedy S, Yazici Y (2025) Evaluation of safety and efficacy of a single Lorecivivint injection in patients with knee osteoarthritis: a multicenter. Obs Ext Trial Rheumatol Ther 12(1):157–171. 10.1007/s40744-024-00731-9 10.1007/s40744-024-00731-9 PMC11751338 39755925

[CR189] Szponder T, Latalski M, Danielewicz A, Krac K, Kozera A, Drzewiecka B, Nguyen Ngoc D, Dobko D, Wessely-Szponder J (2022) Osteoarthritis: pathogenesis, animal models, and new regenerative therapies. J Clin Med. 10.3390/jcm12010005 36614806 10.3390/jcm12010005 PMC9821671

[CR190] Szwedowski D, Szczepanek J, Paczesny L, Zabrzynski J, Gagat M, Mobasheri A, Jeka S (2021) The effect of Platelet-Rich plasma on the intra-articular microenvironment in knee Osteoarthritis. Int J Mol Sci. 10.3390/ijms22115492 34071037 10.3390/ijms22115492 PMC8197096

[CR191] Tambiah JRS, Kennedy S, Swearingen CJ, Simsek I, Yazici Y, Farr J, Conaghan PG (2021) Individual participant symptom responses to intra-articular Lorecivivint in knee Osteoarthritis: post hoc analysis of a phase 2B trial. Rheumatol Ther 8(2):973–985. 10.1007/s40744-021-00316-w 34101138 10.1007/s40744-021-00316-w PMC8217418

[CR192] Tambiah J, Kennedy S, Swearingen C, Mcalindon T, Yazici Y (2024) Impact of structural severity on outcomes in knee osteoarthritis: an analysis of data from phase 2 and phase 3 Lorecivivint clinical trials. Rheumatology (Oxford). 10.1093/rheumatology/keae610 10.1093/rheumatology/keae610 39495154

[CR193] Tao W, Concepcion AN, Vianen M, Marijnissen ACA, Lafeber F, Radstake T, Pandit A (2021) Multiomics and machine learning accurately predict clinical response to Adalimumab and Etanercept therapy in patients with Rheumatoid Arthritis. Arthritis Rheumatol 73(2):212–222. 10.1002/art.41516 32909363 10.1002/art.41516 PMC7898388

[CR194] Terkawi MA, Ebata T, Yokota S, Takahashi D, Endo T, Matsumae G, Shimizu T, Kadoya K, Iwasaki N (2022) Low-grade inflammation in the pathogenesis of osteoarthritis: cellular and molecular mechanisms and strategies for future therapeutic intervention. Biomedicines. 10.3390/biomedicines10051109 35625846 10.3390/biomedicines10051109 PMC9139060

[CR195] Tudorachi NB, Totu EE, Fifere A, Ardeleanu V, Mocanu V, Mircea C, Isildak I, Smilkov K, Carausu EM (2021) The implication of reactive oxygen species and antioxidants in knee osteoarthritis. Antioxidants (Basel). 10.3390/antiox10060985 34205576 10.3390/antiox10060985 PMC8233827

[CR196] Valerio MS, Edwards JB, Dolan CP, Motherwell JM, Potter BK, Dearth CL, Goldman SM (2023) Effect of targeted cytokine inhibition on progression of post-traumatic osteoarthritis following intra-articular fracture. Int J Mol Sci. 10.3390/ijms241713606 37686412 10.3390/ijms241713606 PMC10487447

[CR197] Van Der Aar E, Deckx H, Dupont S, Fieuw A, Delage S, Larsson S, Struglics A, Lohmander LS, Lalande A, Leroux E, Amantini D, Passier P (2022) Safety, pharmacokinetics, and pharmacodynamics of the ADAMTS-5 inhibitor GLPG1972/S201086 in healthy volunteers and participants with osteoarthritis of the knee or hip. Clin Pharmacol Drug Dev 11(1):112–122. 10.1002/cpdd.1042 34859612 10.1002/cpdd.1042 PMC9299907

[CR198] Van Spil WE, Bierma-Zeinstra SMA, Deveza LA, Arden NK, Bay-Jensen AC, Kraus VB, Carlesso L, Christensen R, Van Der Esch M, Kent P, Knoop J, Ladel C, Little CB, Loeser RF, Losina E, Mills K, Mobasheri A, Nelson AE, Neogi T, Peat GM, Rat AC, Steultjens M, Thomas MJ, Valdes AM, Hunter DJ (2020) A consensus-based framework for conducting and reporting osteoarthritis phenotype research. Arthritis Res Ther 22(1):54. 10.1186/s13075-020-2143-0 32192519 10.1186/s13075-020-2143-0 PMC7083005

[CR199] Vincent TL (2019) IL-1 in osteoarthritis time for a critical review of the literature. Research. 10.12688/f1000research.18831.1 10.12688/f1000research.18831.1 PMC6589928 31249675

[CR200] Wang Z, Huang J, Xie D, He D, Lu A, Liang C (2021) Toward overcoming treatment failure in rheumatoid arthritis. Front Immunol 12:755844. 10.3389/fimmu.2021.755844 35003068 10.3389/fimmu.2021.755844 PMC8732378

[CR201] Watt FE, Wise EM (2021) Osteoarthritis and associated comorbidities: new answers and more questions. Rheumatology (Oxford) 60(9):3966–3968. 10.1093/rheumatology/keab405 33944908 10.1093/rheumatology/keab405

[CR202] Weng Q, Chen Q, Jiang T, Zhang Y, Zhang W, Doherty M, Xie J, Liu K, Li J, Yang T, Wei J, Lei G, Zeng C (2024) Global burden of early-onset osteoarthritis, 1990–2019: results from the Global Burden of Disease Study 2019. Ann Rheum Dis 83(7):915–925. 10.1136/ard-2023-225324 38429104 10.1136/ard-2023-225324

[CR203] Werdyani S, Liu M, Zhang H, Sun G, Furey A, Randell EW, Rahman P, Zhai G (2021) Endotypes of primary osteoarthritis identified by plasma metabolomics analysis. Rheumatology (Oxford) 60(6):2735–2744. 10.1093/rheumatology/keaa693 33159799 10.1093/rheumatology/keaa693 PMC8213424

[CR204] Wijesinghe SN, Badoume A, Nanus DE, Sharma-Oates A, Farah H, Certo M, Alnajjar F, Davis ET, Mauro C, Lindsay MA, Jones SW (2023) Obesity defined molecular endotypes in the synovium of patients with osteoarthritis provides a rationale for therapeutic targeting of fibroblast subsets. Clin Transl Med 13(4):e1232. 10.1002/ctm2.1232 37006170 10.1002/ctm2.1232 PMC10068310

[CR205] Xu Z, Li S, Rozewicki J, Yamashita K, Teraguchi S, Inoue T, Shinnakasu R, Leach S, Kurosaki T, Standley DM (2019) Functional clustering of B cell receptors using sequence and structural features. Mol Syst des Eng 4(4):769–778. 10.1039/C9ME00021F

[CR206] Yazici Y, Mcalindon TE, Fleischmann R, Gibofsky A, Lane NE, Kivitz AJ, Skrepnik N, Armas E, Swearingen CJ, Difrancesco A, Tambiah JRS, Hood J, Hochberg MC (2017) A novel Wnt pathway inhibitor, SM04690, for the treatment of moderate to severe osteoarthritis of the knee: results of a 24-week, randomized, controlled, phase 1 study. Osteoarthr Cartil 25(10):1598–1606. 10.1016/j.joca.2017.07.006 10.1016/j.joca.2017.07.006 28711582

[CR207] Yazici Y, Mcalindon TE, Gibofsky A, Lane NE, Clauw D, Jones M, Bergfeld J, Swearingen CJ, Difrancesco A, Simsek I, Tambiah J, Hochberg MC (2020) Lorecivivint, a Novel Intraarticular CDC-like Kinase 2 and dual-specificity tyrosine phosphorylation-regulated kinase 1a inhibitor and wnt pathway modulator for the treatment of knee osteoarthritis: a phase II randomized trial. Arthritis Rheumatol 72(10):1694–1706. 10.1002/art.41315 32432388 10.1002/art.41315 PMC7589351

[CR208] Yazici Y, Mcalindon TE, Gibofsky A, Lane NE, Lattermann C, Skrepnik N, Swearingen CJ, Simsek I, Ghandehari H, Difrancesco A, Gibbs J, Tambiah JRS, Hochberg MC (2021) A Phase 2b randomized trial of lorecivivint, a novel intra-articular CLK2/DYRK1A inhibitor and Wnt pathway modulator for knee osteoarthritis. Osteoarthr Cartil 29(5):654–666. 10.1016/j.joca.2021.02.004 10.1016/j.joca.2021.02.004 33588087

[CR209] Yeater TD, Griffith JL, Cruz CJ, Patterson FM, Aldrich JL, Allen KD (2023) Hypertension contributes to exacerbated osteoarthritis pathophysiology in rats in a sex-dependent manner. Arthritis Res Ther 25(1):7. 10.1186/s13075-022-02966-9 36635774 10.1186/s13075-022-02966-9 PMC9835335

[CR210] Yeh YT, Liang CC, Chang CL, Hsu CY, Li PC (2020) Increased risk of knee osteoarthritis in patients using oral N-acetylcysteine: a nationwide cohort study. BMC Musculoskelet Disord 21(1):531. 10.1186/s12891-020-03562-1 32778089 10.1186/s12891-020-03562-1 PMC7418329

[CR211] Yu H, Huang T, Lu WW, Tong L, Chen D (2022) Osteoarthritis Pain. Int J Mol Sci 23(9):4642. 10.3390/ijms23094642 35563035 10.3390/ijms23094642 PMC9105801

[CR212] Yuan C, Pan Z, Zhao K, Li J, Sheng Z, Yao X, Liu H, Zhang X, Yang Y, Yu D, Zhang Y, Xu Y, Zhang ZY, Huang T, Liu W, Ouyang H (2020) Classification of four distinct osteoarthritis subtypes with a knee joint tissue transcriptome atlas. Bone Res 8(1):38. 10.1038/s41413-020-00109-xW 33298863 10.1038/s41413-020-00109-x PMC7658991

[CR213] Zahan OM, Serban O, Gherman C, Fodor D (2020) The evaluation of oxidative stress in osteoarthritis. Med Pharm Rep 93(1):12–22. 10.15386/mpr-1422 32133442 10.15386/mpr-1422 PMC7051818

[CR214] Zeng D, Chen Y, Liao Z, Wei G, Huang X, Liang R, Lu WW, Yi D, Chen Y (2023) Cartilage organoids and osteoarthritis research: a narrative review. Front Bioeng Biotechnol 11:1278692. 10.3389/fbioe.2023.1278692 38026876 10.3389/fbioe.2023.1278692 PMC10666186

[CR215] Zhang Q, Lv H, Chen A, Liu F, Wu X (2012) Efficacy of infliximab in a rabbit model of osteoarthritis. Connect Tissue Res 53(5):355–358. 10.3109/03008207.2012.661001 22288847 10.3109/03008207.2012.661001

[CR216] Zhang XX, He SH, Liang X, Li W, Li TF, Li DF (2021) Aging, cell senescence, the pathogenesis and targeted therapies of osteoarthritis. Front Pharmacol 12:728100. 10.3389/fphar.2021.728100 34497523 10.3389/fphar.2021.728100 PMC8419276

[CR217] Zhang Y, Ren J, Zang Y, Guo W, Disantis A, Martin RL (2023) Cross-culturally adapted versions of patient reported outcome measures for the lower extremity. Int J Sports Phys Ther V18(3):653–686. 10.26603/001c.74528 37425110 10.26603/001c.74528 PMC10324371

[CR218] Zhao J, Huang H, Liang G, Zeng LF, Yang W, Liu J (2020) Effects and safety of the combination of platelet-rich plasma (PRP) and hyaluronic acid (HA) in the treatment of knee osteoarthritis: a systematic review and meta-analysis. BMC Musculoskelet Disord 21(1):224. 10.1186/s12891-020-03262-w 32278352 10.1186/s12891-020-03262-w PMC7149899

[CR219] Zheng W, Feng Z, You S, Zhang H, Tao Z, Wang Q, Chen H, Wu Y (2017) Fisetin inhibits IL-1beta-induced inflammatory response in human osteoarthritis chondrocytes through activating SIRT1 and attenuates the progression of osteoarthritis in mice. Int Immunopharmacol 45:135–147. 10.1016/j.intimp.2017.02.009 28213268 10.1016/j.intimp.2017.02.009

